# Tectonic collision and uplift of Wallacea triggered the global songbird radiation

**DOI:** 10.1038/ncomms12709

**Published:** 2016-08-30

**Authors:** Robert G. Moyle, Carl H. Oliveros, Michael J. Andersen, Peter A. Hosner, Brett W. Benz, Joseph D. Manthey, Scott L. Travers, Rafe M. Brown, Brant C. Faircloth

**Affiliations:** 1 Biodiversity Institute and Department of Ecology and Evolutionary Biology, University of Kansas, Lawrence, Kansas 66045, USA; 2 Department of Biology and Museum of Southwestern Biology, University of New Mexico, Albuquerque, New Mexico 87131, USA; 3 Department of Biology, University of Florida, Gainesville, Florida 32611, USA; 4 Division of Vertebrate Zoology, American Museum of Natural History, New York, New York 10024, USA; 5 Department of Biological Sciences and Museum of Natural Science, Louisiana State University, Baton Rouge, Louisiana 70803, USA

## Abstract

Songbirds (oscine passerines) are the most species-rich and cosmopolitan bird group, comprising almost half of global avian diversity. Songbirds originated in Australia, but the evolutionary trajectory from a single species in an isolated continent to worldwide proliferation is poorly understood. Here, we combine the first comprehensive genome-scale DNA sequence data set for songbirds, fossil-based time calibrations, and geologically informed biogeographic reconstructions to provide a well-supported evolutionary hypothesis for the group. We show that songbird diversification began in the Oligocene, but accelerated in the early Miocene, at approximately half the age of most previous estimates. This burst of diversification occurred coincident with extensive island formation in Wallacea, which provided the first dispersal corridor out of Australia, and resulted in independent waves of songbird expansion through Asia to the rest of the globe. Our results reconcile songbird evolution with Earth history and link a major radiation of terrestrial biodiversity to early diversification within an isolated Australian continent.

## Abstract (web summary)

Songbirds originated in Australia and have now diversified into approximately 5,000 species found across the world. Here, Moyle *et al*. combine phylogenomic and biogeographic analyses to show that songbird diversification was associated with the formation of islands providing a route out of Australia.

## Graphical abstract

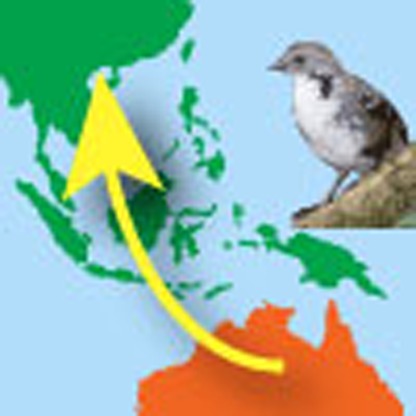

Avian diversity is concentrated conspicuously in a single clade, the oscine passerines (songbirds; ∼5,000 species), which constitute a major component of avifaunas around the world^1^. Early molecular phylogenetic studies and the fossil record support the hypothesis that songbirds originated in Australia^2^,^3^,^4^,^5^, but most other aspects of early songbird evolution remain uncertain^6^. Prior research suggested songbird diversification scenarios that are largely uncoupled from Earth history, including extensive diversification of lineages in New Guinea before its emergence as a landmass^7^,^8^ and long-distance dispersal to Africa or Asia when no dispersal corridors existed^2^,^7^,^9^,^10^,^11^ (but see refs 12, 13, 14). However, these inferences may be compromised by reliance on unresolved phylogenetic relationships and a controversial biogeographic time calibration. Molecular phylogenetic studies have identified major lineages of songbirds, but these studies also revealed rapid, early bursts of diversification that have proven intractable to all analyses^2^,^9^,^15^. Studies using the largest multi-gene data sets could not resolve many relationships within two major songbird radiations, the Corvides and Passerides^2^,^8^,^16^,^17^ (sensu Cracraft^18^). For example, the most extensive character sampling to date yielded songbird phylogenies with low support (that is, <70% bootstrap support) for >50% of nodes^8^,^15^. Two major genome-scale phylogenetic studies of birds included only a few representatives from the oscine passerines^19^,^20^.

Attempts to understand the evolutionary history of songbirds have also been hampered by a sparse fossil record that provides few clues about the age and geographic origin of major clades^5^. Revelation of a likely Gondwanan origin for all passerine birds^3^,^4^,^21^ initiated a paradigm shift in biogeographic inquiry for the group and enabled an explicit timescale for passerine evolution based on geologic events during the breakup of the Gondwanan landmasses^2^,^3^. Using these biogeographic calibrations and partially resolved molecular phylogenies, studies have identified key events in crown-oscine evolution^2^,^3^,^7^,^9^,^10^, such as (1) a Palaeocene or Eocene origin in Australasia and early diversification of the Corvides among ‘proto-islands' of New Guinea; (2) an African origin of the largest clade of Passerides via dispersal across the Indian Ocean from Australia; and (3) multiple Palaeogene long-distance dispersal events to Asia. However, this set of palaeobiogeographic hypotheses produced an evolutionary chronology incongruent with other aspects of geologic history, such as the Miocene formation of New Guinea and Wallacea^22^,^23^. Recent studies using a variety of fossil calibration points offered an alternative time frame for bird diversification that is substantially younger, but they included few songbirds^19^,^20^ or did not resolve basal songbird relationships^12^,^13^.

To test previous hypotheses of songbird diversification, we collected genome-scale DNA sequence data to reconstruct songbird relationships, and we placed this phylogeny in a temporal context with fossil-derived calibration points. Phylogenetic analysis of these data produced a well-supported hypothesis of relationships among oscines that is highly concordant across multiple analytical approaches. Our biogeographic reconstructions support early songbird diversification in Australia in the Oligocene, followed by rapid diversification and geographic expansion via SE Asia early in the Miocene, coincident with extensive island formation in Wallacea.

## Results

We prepared reduced representation genomic libraries for 104 songbird species (Supplementary Data 1), representing ∼70% of songbird families, using a sequence capture protocol (www.ultraconserved.org) that targeted ultraconserved elements (UCEs)^24^. We obtained an average of 4.7 × 10^6^ reads for each individual after discarding reads with low-quality scores (Supplementary Data 1). Trimming reads with adaptor contamination yielded an average read length of 98.9 bp across all individuals. From these reads, we assembled contigs corresponding to 3,096–4,644 UCE loci (mean=4,286.8) for each individual having an average length of 695.9 bp and average coverage of 47.9 × . As expected, the sample taken from a toepad clipping of a museum specimen (*Mohoua albicilla*) yielded the least amount of data, with the lowest number of UCE loci (3,096) and the lowest average contig length (268.1 bp). Sequence capture yielded 4,155 UCE loci present in at least 75% of taxa and an alignment length of just under 2.5 million bases (incomplete matrix). We also created a stringent data set of 515 loci recovered in all taxa with an aligned length of just over 375,000 bases (complete matrix).

**Figure 1:**
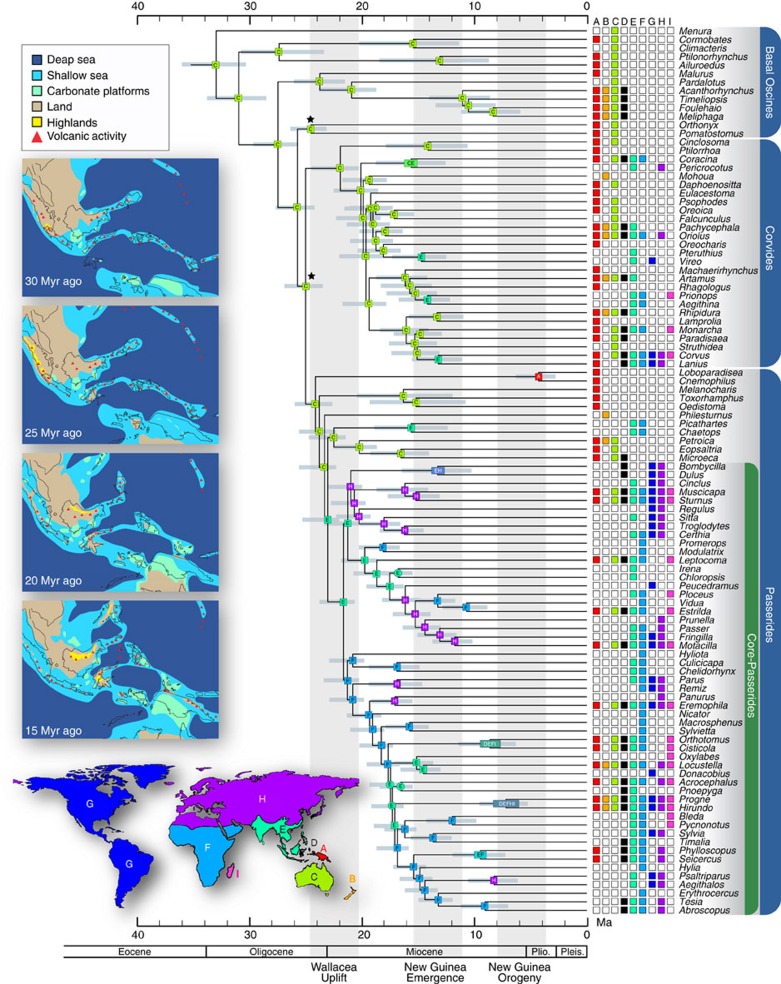
Time-calibrated phylogeny of songbirds. Topology based on concatenated maximum likelihood analysis of 4,155 UCE loci and date estimates based on fossil-derived calibrations. Nodes with squares around ancestral range indicate 70% or greater maximum likelihood bootstrap support, circles indicate <70% bootstrap support. All nodes are supported by posterior probability greater than 0.98 in Bayesian analyses. Geological events are indicated by vertical grey bars and labelled below. Stars indicate nodes calibrated for dating analyses. Coloured squares at tips indicate distribution of clade for that exemplar and correspond to coloured world map (A, Australia; B, New Zealand; C, New Guinea; D, Wallacea; E, S and SE Asia; F, Africa; G, New World; H, Palaearctic; I, Madagascar). Ancestral areas at internal nodes inferred with DEC+*j* model and New Guinea excluded as an ancestral area before 15 Myr ago. Palaeogeographic reconstructions adapted from Hall^27^ depict the distribution of land and sea across Wallacea and northern Australasia during early songbird diversification. Abbreviations: Plio.=Pliocene; Pleis.=Pleistocene. See (Supplementary Fig. 6) for dating with alternative calibrations.

Concatenation and coalescent approaches to species tree inference yielded well-resolved and highly concordant topologies (Fig. 1; Supplementary Figs 1 and 2). Bayesian and maximum likelihood methods produced nearly identical trees and congruent support indices (Supplementary Fig. 1). Of 103 internal nodes, 84 had support of 1.0 posterior probability and 100% bootstrap support. Only six nodes had <70% bootstrap support, but still had high posterior probability (>0.98). SVDquartets recovered a topology highly congruent with Bayesian and maximum likelihood estimates with only 12 nodes receiving <70% bootstrap support (Supplementary Fig. 2). Gene tree-based coalescent methods recovered trees with lower nodal support and were affected by bias caused by missing data (Supplementary Figs 3 and 4, Supplementary Discussion). Our results differed markedly from previous studies (Supplementary Fig. 5). As an example, at the base of the Passerides we found strong support for sequential branching of the New Guinea endemic Cnemophilidae and Melanocharitidae followed by the New Zealand endemic Callaeidae and then a sister pairing of the Picathartidae (Africa and Asia) and Petroicidae (Australasia). These families have been placed in various configurations near the Passerides and Corvides in previous studies^2^,^7^,^9^,^13^,^15^ but their unanticipated affinities require reinterpretation of previous biogeographic hypotheses.

A relaxed clock analysis of the complete matrix using the earliest fossil identifiable as a crown-oscine^25^ and secondary calibrations derived from a fossil-calibrated phylogenomic analysis of birds^19^ produced a time frame (Fig. 1) more recent than hypothesized by most studies (but see refs 12, 13, 20). The first songbird lineages diverged in the Oligocene (basal oscines; Fig. 1), but the vast majority of major clades diversified in the Miocene. A large concentration of short internodes in multiple clades of the Corvides and Passerides cluster at 13–20 Myr ago. An alternative dating analysis based on secondary calibrations from a more recent study^20^ yielded slightly older date estimates (Supplementary Fig. 6).

Biogeographic analyses using geologically informed constraints overwhelmingly support early songbird diversification in Australia (Fig. 1; Supplementary Figs 7–10). Early diversification in the Corvides and Passerides also occurred within Australia in the Miocene, in contrast to previous findings that the Corvides arose and diversified within proto-islands of New Guinea in the Eocene^7^ or Oligocene^8^. Our biogeographic results derive from the incorporation of recent geological models for the formation of New Guinea^23^,^26^ (see Supplementary Discussion) as a constraint on the biogeographic analysis (that is, New Guinea was not allowed as an ancestral range before its existence as a landmass). The exact timing and details of competing models differ, but all agree that New Guinea emerged as a substantial island no earlier than 12 Myr ago^23^, and possibly as late as 5 Myr ago^26^. Unconstrained analyses (Supplementary Fig. 7) imply New Guinea origins for nodes much deeper in the tree^7^,^8^ but these are inconsistent with the geological record.

## Discussion

Our biogeographic analyses indicate that all dispersal out of the Australian region, beginning at *ca*. 23 Myr ago, occurred via Asia followed by rapid colonization of other continents (Fig. 1). An early lineage of Passerides dispersed to Asia *ca*. 24 Myr ago, followed by several lineages of Corvides and Passerides starting *ca*. 16 Myr ago. This rapid colonization and diversification produced the majority of short internodes that plagued previous analyses. The time frame of dispersal out of Australia is consistent with the Sula spur of Australia colliding with proto-Sulawesi in the early Miocene, an event which initiated substantial island formation in Wallacea and provided the first links from Australasia to Sundaland^22^,^27^. Subsidence of newly formed land in Wallacea by 15 Myr ago reduced the dispersal corridor and may explain the lack of relictual endemics in the region^22^. Previous studies using biogeographic calibrations inferred this cluster of dispersal events to occur in the Eocene and Oligocene^2^,^7^ when Australia was isolated by thousands of kilometres of open ocean.

A recently published timescale for avian diversification^12^ obtained node ages within songbirds that were substantially more recent than most previous studies, yet older than our timing estimates. Although their study also concluded that the more recent time frame of songbird diversification obviates the need to invoke long-distance dispersal to explain the expansion of songbirds out of the Australian region, they did not provide an explicit biogeographic hypothesis of how ‘oscine lineages were transferred' to Southeast Asia during the late Eocene to Oligocene; perhaps because these continental regions were still separated by a substantial marine gap and deep water trench during this period^22^,^28^. Moreover, the microplate hypothesis which they cite as a possible mechanism for transporting Australian continental fragments (and presumably songbird lineages) west to Wallacea in the Miocene–Pliocene is now considered implausible based on the most recent palaeogeological modelling^22^. Their biogeographic results are similar to ours despite substantial disparity in phylogenetic relationships compared with our results. Although the backbone of the phylogeny they presented used published genomic data^19^, all oscine relationships were reconstructed with two protein-coding nuclear genes and node support was not given. Their results also indicated substantial diversification in Australia before dispersal to Asia, but New Guinea was not coded as a separate area, thus these results are not directly comparable to our biogeographic results.

A recent hypothesis for songbird diversification identified New Guinea as an ancestral range for many nodes deep in the songbird tree, especially within the Corvides^7^. These nodes were dated as Eocene to Oligocene in age, long before New Guinea existed as a substantial landmass, and so ‘proto-Papuan' islands were identified as the source of substantial early diversification of the Corvides. Our ancestral range analyses that were naive to the existence of the New Guinea landmass corroborate these biogeographic results, but our analyses that factor in the timing of New Guinea formation support an alternative hypothesis, namely that most early diversification occurred within Australia but many of these Australian lineages disappeared and/or dispersed to New Guinea more recently^29^,^30^. We believe that unconstrained biogeographic analyses are biased by extinctions caused by the extensive aridification of Australia (Supplementary Discussion). This aridification, which began in the Miocene but continued throughout the uplift of New Guinea, likely caused many wet-forest-adapted songbirds in Australia to become extinct or restricted to wet forest habitats in New Guinea, as has been documented in other taxa^31^. For example, southern beech (subgenus *Brassospora*) that once occurred in formerly widespread Australian wet forests is now absent from Australia, but it remains a diverse component of the montane wet forests in New Guinea (see Supplementary Discussion for additional examples). Both hypotheses invoke substantial assumptions about diversification processes (for example, origin of a major avian clade and substantial extant diversity on ephemeral islands versus substantial extinction and range shifts); however, our data provide support for the hypothesis that New Guinea played no role in early songbird diversification, but instead was a region of major subsequent diversification of relictual, wet-forest-adapted Australian lineages^29^.

Several studies have advocated an African origin of the core-Passerides, resulting from direct colonization of Africa from Australasia rather than via Asia^10^,^32^. This hypothesis is based on a perceived preponderance of African taxa branching from the base of the passeridan clade, and an early time frame of diversification that allowed a dispersal scenario across now-submerged landmasses and archipelagoes in the Southern Indian Ocean. Our phylogeny, time estimates, and biogeographic reconstruction cast doubt on this dispersal route. After *ca*. 43 Myr ago, any dispersal across the southern Indian Ocean became increasingly unlikely, because accelerated spreading at the Southeast Indian Ridge sundered Broken Ridge, part of a putative dispersal corridor across the southern Indian Ocean^29^, and propelled Australia towards Sundaland^33^. Rather, our proposed diversification chronology supports the more traditional dispersal route through Asia for the Passerides^2^,^4^. Our estimates place all diversification and dispersal of the Passerides in the Miocene, contemporary with extensive island formation in Wallacea^22^ that provided a direct route of colonization from Australasia to Asia.

Specific phylogenetic relationships also cast doubt on direct dispersal to Africa. Previous studies reconstructed Picathartidae as sister to the core-Passerides^9^,^10^ or Corvides^12^. However, we found strong support that Picathartidae is sister to Petroicidae, an Australasian family; uncertainty regarding relationships within Picathartidae render its origin ambiguous^15^. Thus, Picathartidae provides no evidence for an African origin of core-Passerides, unless we also assume that the ancestor of the Petroicidae re-crossed the southern Indian Ocean to re-colonize Australasia. The African endemic *Hyliota* has also been cited as evidence of an African origin of the core-Passerides^32^, yet this inference relied on an unresolved position near the base of the Passerides. We do not recover *Hyliota* at the base of the core-Passerides—rather it is the sister lineage to the Stenostiridae, a family found in Africa and Asia. Thus, *Hyliota* also provides no direct evidence of an African origin for core-Passerides.

Our study provides a well-resolved phylogeny of songbirds, clarifies the relationships of all major lineages, and provides a time frame of evolution that implicates key geological drivers of diversification. Songbirds originated in the Eocene but diversified within an isolated Australian continent in the Oligocene. Tectonic collision and uplift of Wallacea as Australia drifted north allowed dispersal to Asia and proliferation of songbird species across the rest of the world, where most of their diversity now occurs. Although New Guinea presently contains high songbird species richness, our data provide an alternative to the proto-Papuan hypothesis for early songbird diversification^7^,^8^ and suggest that New Guinea has, instead, served as an ‘evolutionary refuge' for Australian lineages that have diversified more recently within the island^29^. Substantial songbird diversity in New Guinea likely developed recently in response to Papuan orogeny, consistent with diversification patterns for other groups in New Guinea^34^ and worldwide^35^,^36^.

## Methods

### Laboratory techniques

We extracted and purified DNA from fresh muscle or liver tissue, or toepad clips from museum specimens using the Qiagen DNeasy Blood and Tissue Kit following the manufacturer's protocol. We quantified DNA extracts using a Qubit 2.0 Fluorometer, and we sheared 500 ng of DNA of each sample (except for one sample extracted from the toepad of a museum study skin, which was not sheared) to 400–600 bp in 50 μl volume using a Covaris S220 sonicator at 175 W peak incident power, 2% duty factor, and 200 cycles per burst for 45 s. We performed ¼ reactions of end repair, A-tailing, and adaptor ligation on Solid Phase Reversible Immobilization beads using Kapa Biosystems Library Prep kits following the procedure of Faircloth *et al*.^24^ (described in detail at http://ultraconserved.org). We ligated universal iTru stubs^37^ instead of sample-specific adaptors to allow for dual indexing, and added a second AMPure XP bead clean up at 1.0 × volume after stub ligation. We incorporated iTru dual-indexes^37^ to library fragments using a 17-cycle PCR with NEB Phusion High-Fidelity PCR Master Mix.

We quantified libraries using a Qubit 2.0 Fluorometer and pooled sets of eight samples at equimolar ratios for enrichment. We performed sequence capture and post-enrichment amplification following standard protocols^24^ using the Mycroarray MYbaits kit for Tetrapods UCE 5K version 1, which targets 5,060 UCE loci. This procedure involved hybridizing biotinylated RNA probes with pooled libraries for 24 h, after which we enriched DNA targets using streptavidin-coated beads. We amplified fragments with a 17-cycle PCR amplification step using NEB Phusion High-Fidelity PCR Master Mix. After post-enrichment amplification, we quantified libraries using an Illumina Eco qPCR System and a commercial library quantification kit (Kapa Biosystems), combined 96 libraries into a 10 μM pool, and sequenced the pooled libraries in a high output, paired-end run of 100 cycles on an Illumina HiSeq 2500 System at the University of Kansas Genome Sequencing Core.

### Data assembly

We de-multiplexed raw reads using CASAVA ver. 1.8.2 and trimmed low-quality bases and adaptor sequences from reads using illumiprocessor ver. 1 (https://github.com/faircloth-lab/illumiprocessor) which batch processed reads using Scythe (https://github.com/vsbuffalo/scythe) and Sickle (https://github.com/najoshi/sickle). We used the Python package PHYLUCE^38^ for subsequent data processing. We assembled cleaned reads into contigs using Trinity ver. r2013.08.14 (ref. 39) and extracted contigs for each taxon that matched UCE loci. We assembled an incomplete data set containing UCE loci that were present in at least 75% of all 106 taxa for maximum likelihood (ML), Bayesian (BI), and SVDquartets^40^ analyses. For gene tree-based species tree analyses, we assembled complete data sets consisting of loci common to all taxa being analysed. For each data set, we aligned each locus using MAFFT^41^, and we trimmed resulting alignments to allow missing nucleotides at the flanks of each alignment only if at least 65% of taxa contained data, which is the default option in PHYLUCE. We further trimmed uncertain alignment regions using Gblocks^42^ with default parameters except for the minimum number of sequences for a flank position in Gblocks, which we set at 65% of taxa.

Protein-coding genes can provide convergent signal and base-composition bias in phylogenomic data sets^19^. We undertook two approaches to address this potential concern. First, using BEDTools^43^ we aligned the UCE probes to known chicken protein-coding genes and identified UCE loci whose probe(s)+300 bp of flanking region overlap with these genes. Of the 4,155 UCE loci recovered in the incomplete matrix, only 316 (7.6%) overlapped with chicken protein-coding genes. We assembled a data set without these 316 loci. Second, we recoded the incomplete matrix as purines and pyrimidines (RY-coding). We analysed both data sets with a maximum likelihood approach (Supplementary Fig. 11; see below).

### Phylogenetic analyses

We performed maximum likelihood (ML) inference on the concatenated loci of the incomplete and complete data sets using RAxML ver. 8.1.3 (ref. 44) assuming a general time reversible model of rate substitution and gamma-distributed rates among sites. We evaluated node support using 500 rapid bootstrap replicates. We tested for convergence of bootstrap replicates *a posteriori* using the ‘autoMRE' option in RAxML. For BI, we used Exabayes ver. 1.4.2 (ref. 45) with loci partitioned according to evolutionary model (determined with Cloudforest http://github.com/ngcrawford/cloudforest). We unlinked state frequencies, rate heterogeneity among sites, and the substitution matrix among partitions; we linked branch lengths across partitions. Exabayes analyses tended to converge on single topologies with no subsequent topology swaps accepted when using default settings. To more thoroughly search parameter space, we adjusted the temperature of the heated chains (heatFactor), topology proposals (for example, increase parsimonySPR and likeSPR) and parameters for various moves (parsSPRRadius, parsimonyWarp, likesprmaxradius, and likesprwarp). Changes in these settings often represented a trade-off between more thorough tree searching at the expense of greatly increased computation time, especially increasing the likelihood SPR settings. We eventually converged on settings with more liberal parsimony criteria (for example, radius 10,000, increased parsimony proposals (∼20%), etc.) and moderate likelihood criteria (for example, radius 10, modest likelihood proposal frequency (∼5%), etc.) that prevented getting stuck in local optima while remaining computationally tractable. All Exabayes analyses included multiple independent runs (2 runs, each with one cold and two heated chains); we assessed convergence between runs with the average standard deviation of split frequencies <0.01, plots of likelihood and parameter estimates, the potential scale reduction factor close to one (<1.1), and effective sampling size (ESS) >200.

To obtain a species tree estimate that did not rely on prior inference of individual gene trees, we used SVDquartets^40^,^46^, a method that analyzes quartets of species in a coalescent framework using singular value decomposition of the matrix of site pattern frequencies and then assembles a species tree from the quartets using a supertree method. This method was developed for analysis of unlinked single nucleotide polymorphism data, but extended to analysis of DNA sequences, especially large, multi-locus data sets. We generated 100 bootstrap replicates of the data using RAxML ver. 8.1.3 (ref. 44). For each bootstrap replicate we generated all quartets using the implementation of SVDquartets in a test version of Paup, ver. 4.0a146 (ref. 47) and then assembled the species tree using the quartet max-cut method^48^. For gene tree-based coalescent methods for species tree analysis, we performed gene-tree inference and bootstrapping with RAxML ver. 8.1.3 (ref. 44) using the Python package PHYLUCE^38^. We modified the PHYLUCE scripts to implement multi-locus bootstrapping^49^ (that is, sampling with replacement of loci and sites), and we generated 500 multi-locus bootstrap replicate sets of gene trees for each data set. On each replicate set of gene trees, we ran four methods using default parameters: STAR and STEAC as implemented in the R package phybase ver. 1.3 (ref. 50), MP-EST ver. 1.4 (ref. 51), and ASTRAL ver. 4.7.7 (ref. 52). ASTRAL is not strictly a coalescent method, but it is statistically consistent with the multispecies coalescent model^52^, and as such, other authors have included it in the family of coalescent-based gene tree methods^53^. Command line, Python, and R scripts used to process the data and run species tree analyses are available from https://github.com/carloliveros/uce-scripts.

### Divergence time estimation

Most previous analyses of passerine diversification^2^,^7^,^8^ used a putative biogeographic event as a calibration for dating (that is, the separation of NZ and Australia at ∼80 Myr ago to date the node separating *Acanthisitta* from all other passerines), but incorporated no geological information in their biogeographic reconstructions. For example, Wallacea and New Guinea are unconstrained and allowed to occur as ancestral areas (distributions) anywhere in the phylogeny, even though these regions formed recently compared with continental areas and the inferred time frame of bird evolution^22^,^23^,^54^. We took the opposite approach for biogeographic analysis, using biogeography independent calibrations derived from fossils for dating and applying geologically informed palaeogeographic models for biogeographic reconstruction.

The fossil record for passerine birds is sparse and many described fossil taxa lack information necessary for use as informative calibration points^55^. A recent genomic study of birds^19^ used fossil calibrations from a variety of bird orders to calibrate the avian phylogeny. The resulting Jarvis *et al*.^19^ tree sparsely sampled passerines (five species) but included nodes shared with our sampling, such as the split between Corvides and Passerides, which we included in our dating analysis as a normal distribution with a mean of 21 Myr ago and 95% confidence interval of 17–26 Myr ago based on their results. Another recent genomic study of birds^20^ produced a timescale similar to, but slightly older than that of Jarvis *et al*.^19^ We also used an alternative calibration (normal distribution with mean 28 Myr ago and 95% confidence interval of 18–38 Myr ago) from Prum *et al*.^20^ for the same node (split between Corvides and Passerides) in separate analyses to test the influence of these different dates. The dates from Jarvis *et al*.^19^ have been criticized as enforcing a lower bound on the phylogeny that was too recent, which might force unrealistically recent dates on the entire phylogeny^56^. However, further analyses with older lower bounds produced almost identical dates for the passerine nodes^57^. Because secondary calibrations are a step removed from fossil calibration data, we also included a calibration based on fossils of the oscine *Orthonyx kaldowinyeri*^25^ for two reasons. First, multiple fossils of this distinctive taxon have been described, alleviating concerns about potential misidentification. Second, these fossils are the earliest identified crown-oscines (Oligo–Miocene), and are from the well-characterized Riversleigh World Heritage Area of Australia, which provides broad comparative context for dating the fossils. For this calibration we used a truncated Cauchy distribution^58^ with minimum age bound of 24 Myr ago and a relatively flat distribution (offset *P*=1.0, scale *c*=5.0). To constrain the base of the tree, we applied a maximum divergence of 45 Myr ago when using the Jarvis *et al*.^19^ secondary calibration and 55 Myr ago when using the Prum *et al*.^20^ secondary calibration. These lower bounds are older than the lower end of the 95% confidence interval of dates for the split between oscines and suboscines in the respective papers. We inferred dated phylogenies with the complete UCE data set, a constrained ML topology, and two different sets of calibration points (*Orthonyx* fossil with either the Jarvis *et al*.^19^ or Prum *et al*.^20^ estimates for the Corvides-Passerides split and bounds on the base of the tree) using MCMCTree in PAML ver. 4.8 (ref. 59). Divergence times were estimated using the independent rates model in MCMCTree and a birth–death process with species sampling. We applied the HKY85+GAMMA model of nucleotide substitution with four rate categories, the most complex model implemented in MCMCTree, but one that accommodates rate variation among nucleotide sites. Inclusion of variable site rates is likely important for age estimation with UCE data because UCEs display a unique pattern of rate variation, with few substitutions in the core UCE region and more substitutions in flanking regions. Because of the size of the data matrix, we used approximate likelihood calculations with branch lengths estimated using the baseml programme of PAML.

### Ancestral range estimation

Our study contained relatively sparse species sampling over a large clade. To simply code the current distribution of each species included in the analysis might bias reconstructions towards regions with greater sampling. For example, we have sampled extensively in New Guinea and Pacific archipelagos, but sparsely in Wallacea. Our choice of clade exemplars would thus be biased towards our sampling regions. To minimize this issue, we considered each species sampled in the phylogeny to be a representative of an entire clade and used two methods for coding areas. First, we coded each terminal based on the total geographic area occupied by the clade it represents. Second, we coded each terminal based on information about the putative origin of its clade from published phylogenetic studies (Supplementary Data 2). We could not reduce most clades to a single area of origin. When a clade's origin was ambiguous, we used the clade's full distribution, and we excluded areas only occupied by species that are deeply embedded inside the clades of interest.

We included nine biogeographic areas in our analyses (New Guinea (A), New Zealand (B), Australia (C), Wallacea (D), S and SE Asia (including Philippines; E), sub-Saharan Africa (F), New World (G), Palaearctic (including N. Africa; H), and, Madagascar (I)). Ancestral range estimation can be informed by realistic palaeogeographic models based on Earth history^60^,^61^. The formation of New Guinea was a critical event in Australasian palaeogeography, and New Guinea has featured prominently in previous biogeographic analysis of oscines^7^,^8^. All recent models for the formation of New Guinea indicate that substantial land area only existed after 15 Myr ago^23^,^54^, or according to one model only after ∼5 Myr ago^26^,^34^. Thus, ancestral range estimates that recover New Guinea at nodes earlier than the mid-Miocene may not accurately reflect palaeogeographic reality. To incorporate palaeogeographic history, we reconstructed ancestral ranges with the constraint that New Guinea was not allowed to occur until 15 Myr ago, as supported by the geologic record. To examine the influence of this constraint, we also ran all analyses naively (that is, with no geographic constraints).

To estimate ancestral ranges and model the geography of songbird diversification, we fitted likelihood versions of the DEC^61^,^62^ (dispersal, extinction and cladogenesis) and DEC+*j* models^63^ to the time-calibrated songbird tree using BioGeoBEARS ver. 0.2.1 (refs 64, 65). Likelihood implementation of DEC (DEC-LIKE) allows two types of anagenetic events: dispersal, or range expansion; and extinction, or range contraction. DEC-LIKE allows two types of cladogenic events. In sympatric events, each daughter species inherits the distribution of the ancestor. This does not imply sympatric speciation *per se*, only that speciation has occurred within the user-defined area. In cladogenic events, each daughter species inherits separate and non-overlapping portions of the ancestor's range^62^,^63^. BioGeoBEARS also implements a modified version of the DEC model that includes a founder-event speciation parameter (*j*, or ‘jump'), which allows one daughter species to inherit the entire range of the ancestor, and the other daughter to disperse to a novel geographic area outside of the ancestor range. This DEC+*j* model may be particularly useful in island systems, where founder-event speciation is expected^63^. We fit DEC-LIKE and DEC-LIKE+*j* under four different conditions: using total clade distributions or inferred clade origin terminal codings, as well as with and without a constraint on New Guinea emergence at 15 Myr ago. For each of these four model conditions, we used the Akaike Information Criterion (AIC) to select if the DEC-LIKE or DEC-LIKE+*j* model best fit the data.

We chose DEC/DEC+*j* as the most appropriate biogeographic models *a priori* based on their reasonable assumptions. However, to explore whether model choice influenced biogeographic interpretations, we also considered two alternate model families, DIVA^66^,^67^ (dispersal-vicariance analysis) and BAYAREA^68^. Similar to the DEC models, DIVA and BAYAREA models allow geographic ranges to evolve through time, but the specific types of events permitted are less appropriate when defined areas are large. Specifically, DIVA does not allow the ‘sympatric subset' area inheritance of the DEC model, and DIVA is more suited to systems where defined areas are too small for divergence within an area. Similarly, BAYAREA does not allow the ‘cladogenic' area inheritance of the DEC model, another unrealistic assumption. Nonetheless, we also fitted DIVA-LIKE, DIVA-LIKE+*j*, BAYAREA-LIKE, BAYAREA-LIKE+*j* in BioGEOBEARS. For these models, we also ran iterations using both total clade distributions and inferred clade origin terminal codings, and we ran iterations with and without a constraint on New Guinea emergence at 15 Myr ago (Supplementary Table 1).

### Data availability

Nucleotide alignments are deposited at Dryad (doi:10.5061/dryad.nf01p), raw sequencing reads at the NCBI SRA (SRA BioSample Nos. SAMN04301695 to SAMN04301800), and UCE nucleotide sequences at NCBI Genbank (Accession Nos. KAAD00000000 to KAEE00000000). Command line, Python, and R scripts are available from https://github.com/carloliveros/uce-scripts. All other data are available from the corresponding author upon request.

## Additional information

**How to cite this article:** Moyle, R. G. *et al*. Tectonic collision and uplift of Wallacea triggered the global songbird radiation. *Nat. Commun.* 7:12709 doi: 10.1038/ncomms12709 (2016).

## Supplementary Material

Supplementary Information Supplementary Figures 1-11, Supplementary Table 1, Supplementary Discussion and Supplementary References

Supplementary Data 1 Sampling information and molecular results.

Supplementary Data 2 Area coding for ancestral range estimation and notes on clade origins.
