# Supplementary material for: Tectonic collision and uplift of Wallacea triggered the global songbird radiation
Source: Nat Commun. 2016 Aug 30;7:12709. doi: 10.1038/ncomms12709 (PMC5013600; doi:10.1038/ncomms12709)
Supplement: Supplementary Information — Supplementary Figures 1-11, Supplementary Table 1, Supplementary Discussion and Supplementary References [file ncomms12709-s1.pdf]

Supplementary Figures

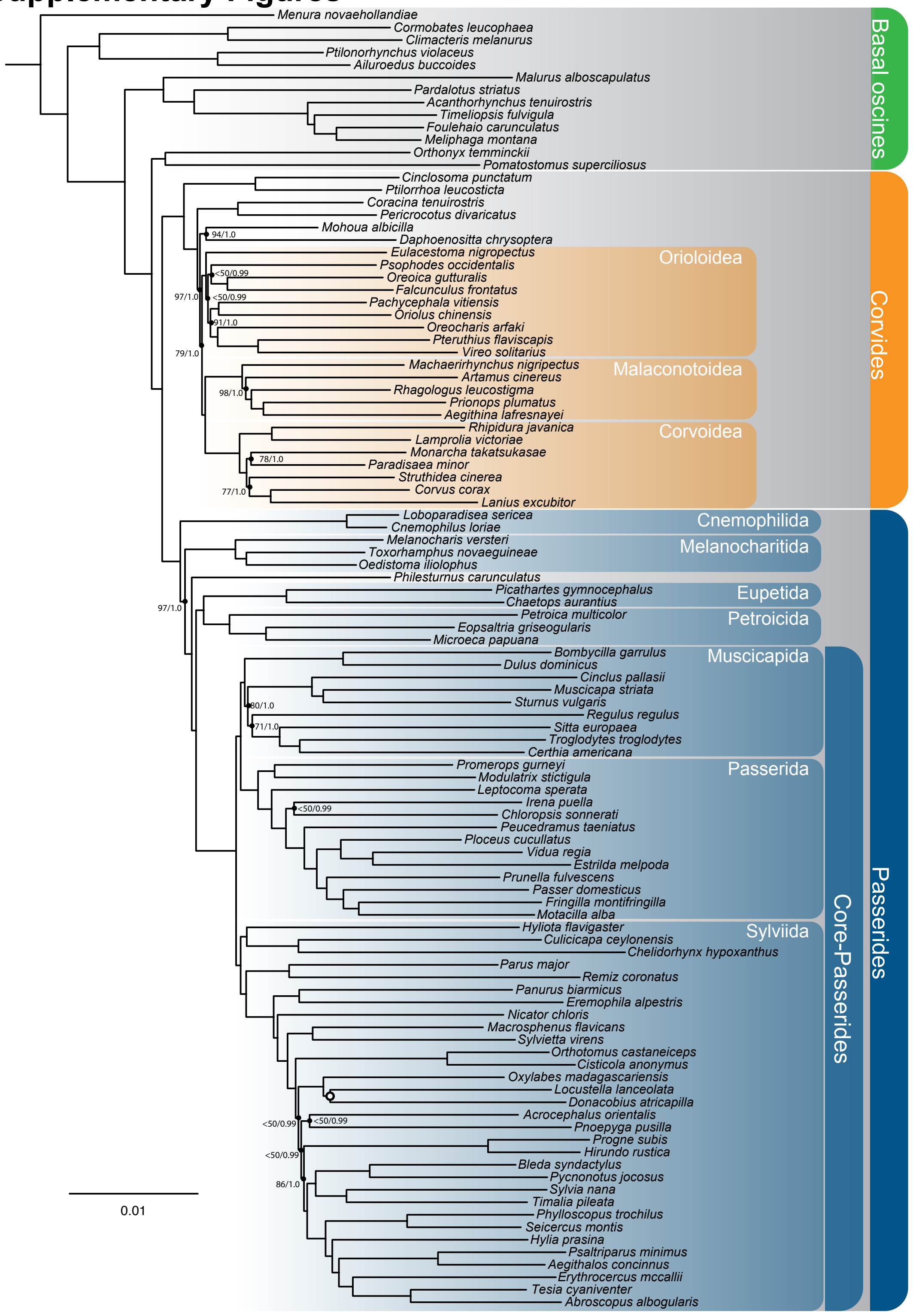

Supplementary Figure 1. Maximum likelihood tree from the incomplete matrix. Numbers by nodes indicate ML bootstrap support/posterior probability from Bayesian analysis. Unlabelled nodes have support of 100/1.0. Circled node indicates the lone discrepancy in concatenated analyses (see text). Sub-clades of Corvides are shown at the superfamily level, whereas Passerides subclades are shown at the parvorder level (see Supplementary Discussion for details).

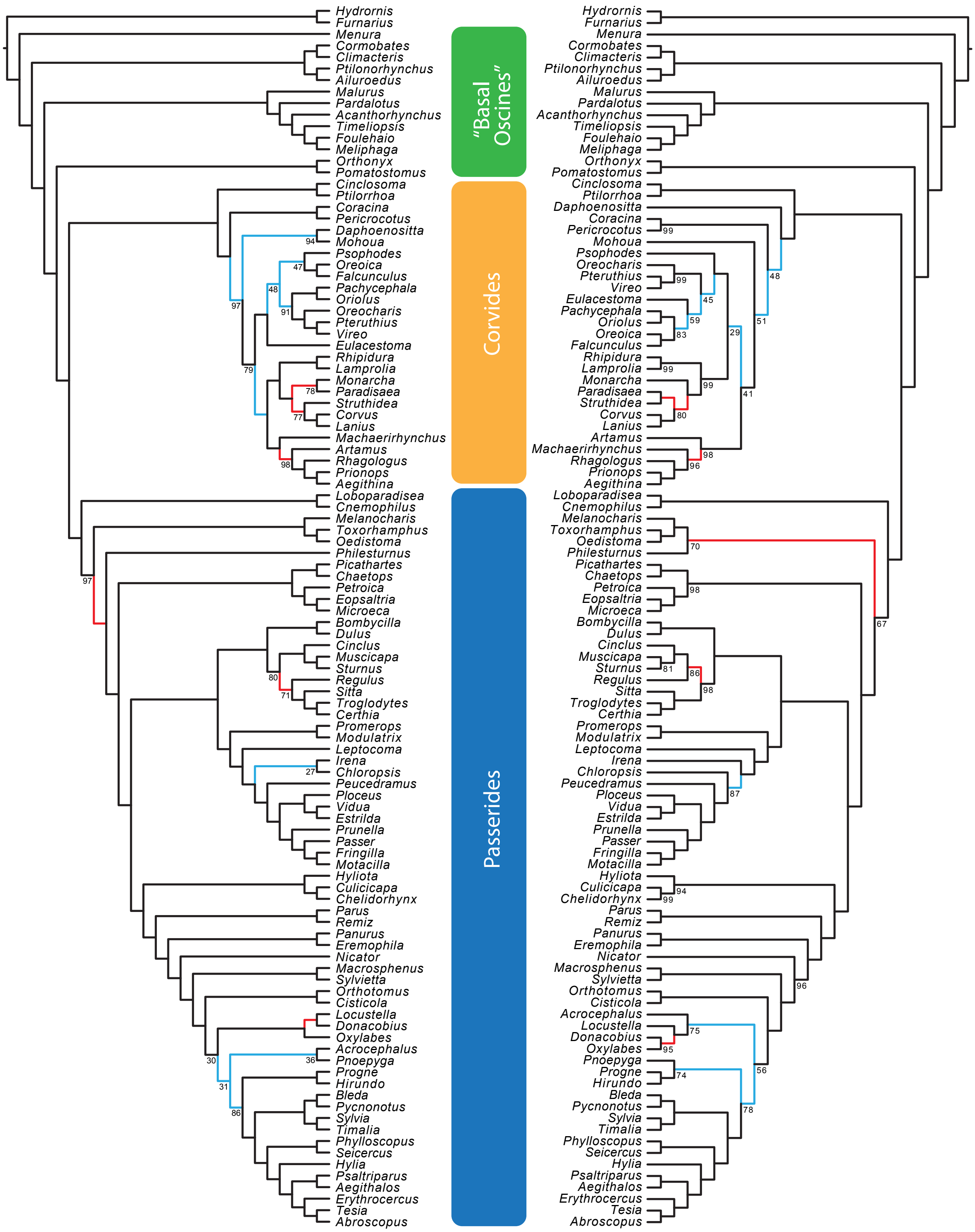

Supplementary Figure 2. Phylogenetic relationships among oscines estimated using concatenation (maximum likelihood estimation with RAxML, left) and coalescent approaches (SVDquartets, right). Bootstrap support values are 100% unless otherwise indicated by numbers below nodes. Red branches indicate conflicting relationships that are highly supported (bootstrap proportion ≥ 70%) in both approaches; blue branches indicate conflicting relationships that are not highly supported by one of the approaches. Taxa involved in conflicting relationships are highlighted in brown.

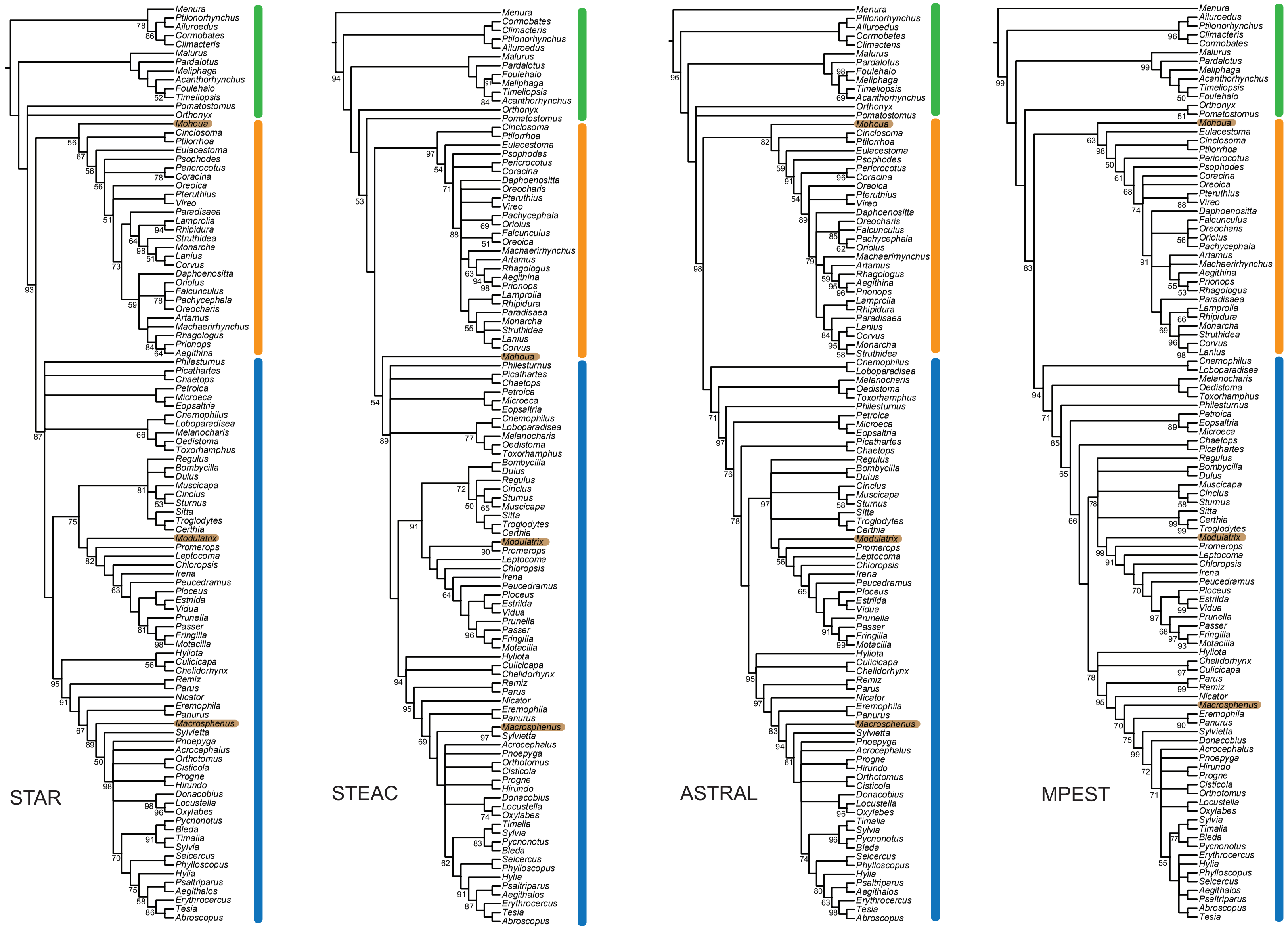

Supplementary Figure 3. Results of gene-tree based coalescent methods. Bootstrap percentages shown by nodes. Nodes with 100% bootstrap support are unlabeled; nodes with less than 50% support collapsed. Highlighted genera indicate samples with short contig lengths as discussed in text. Colored bars indicate basal oscines (green), Corvides (orange), and Passerides (blue).

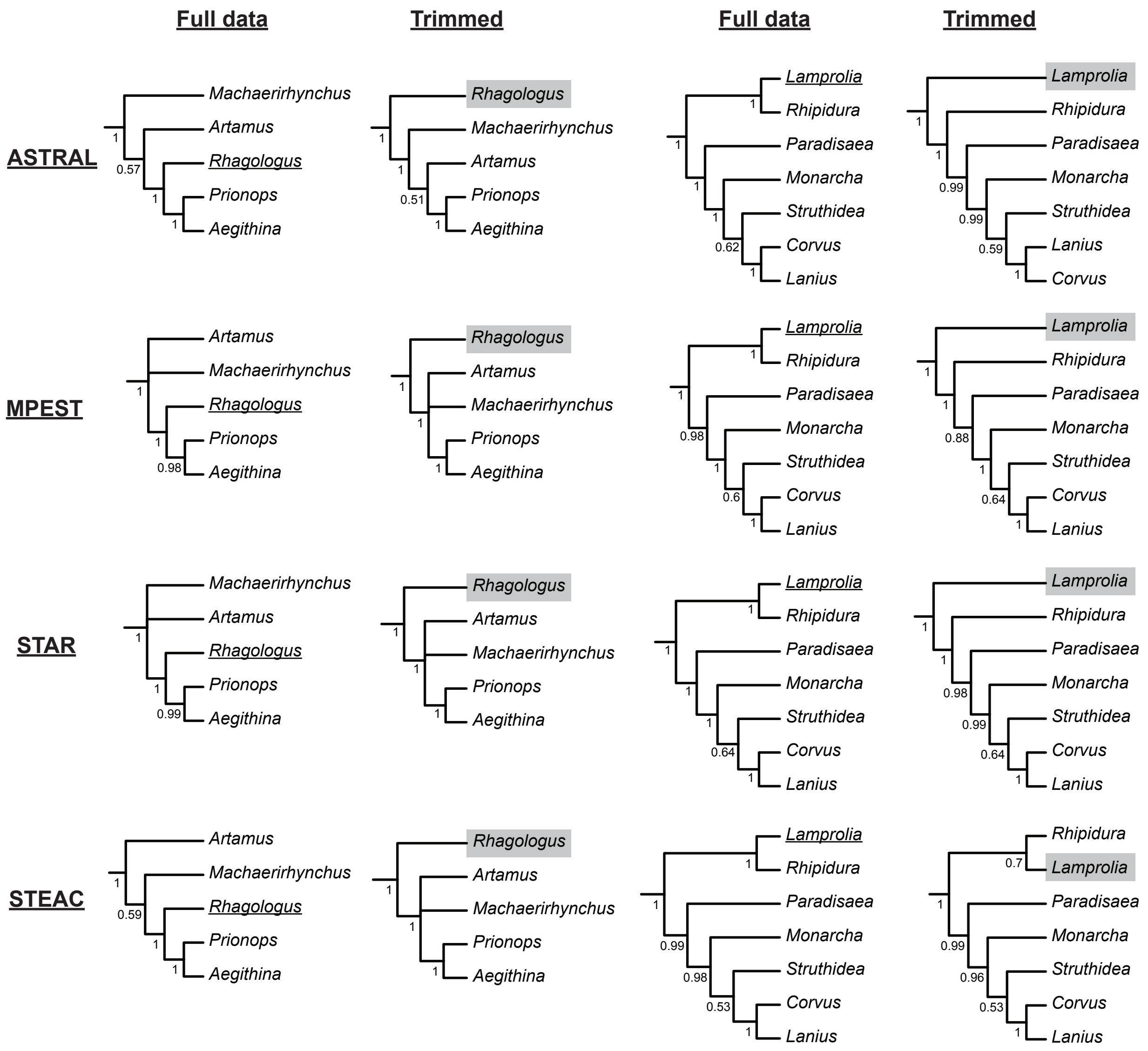

Supplemental Figure 4. Results of GCM analyses with experimental trimming of select sequences to mimic short contigs. Each clade includes analyses of the full data (left) and with one sample (underlined or boxed) trimmed to the same contig lengths as those of *Mohoua* (the shortest contigs in our data set). In each case, the trimmed sample moved to a position closer to the root with high support.

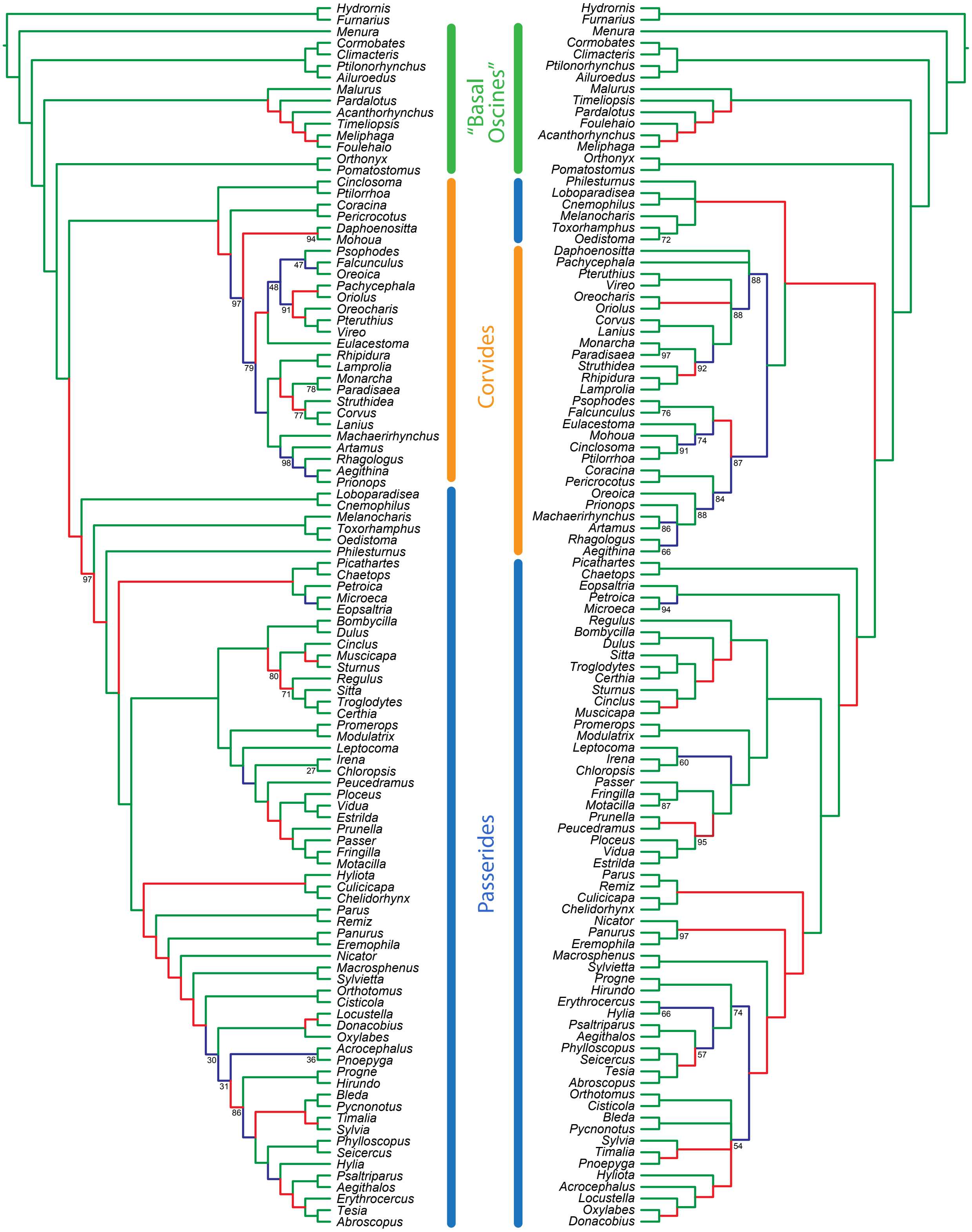

Supplementary Figure. 5. Phylogenetic relationships among oscines estimated using concatenation (maximum likelihood estimation with RAxML, left) from this study and from Jetz et al.[10] (consensus of 1000 trees with subset of taxa chosen to match this study using the Hackett et al. 2008 backbone, right). Bootstrap support values (on our tree) and Bayesian posterior probabilities (on Jetz et al. 2012 tree) are 100% unless otherwise indicated by numbers below nodes. Red branches indicate conflicting relationships that are highly supported in both studies; blue branches indicate conflicting relationships that are not highly supported in one of the studies.

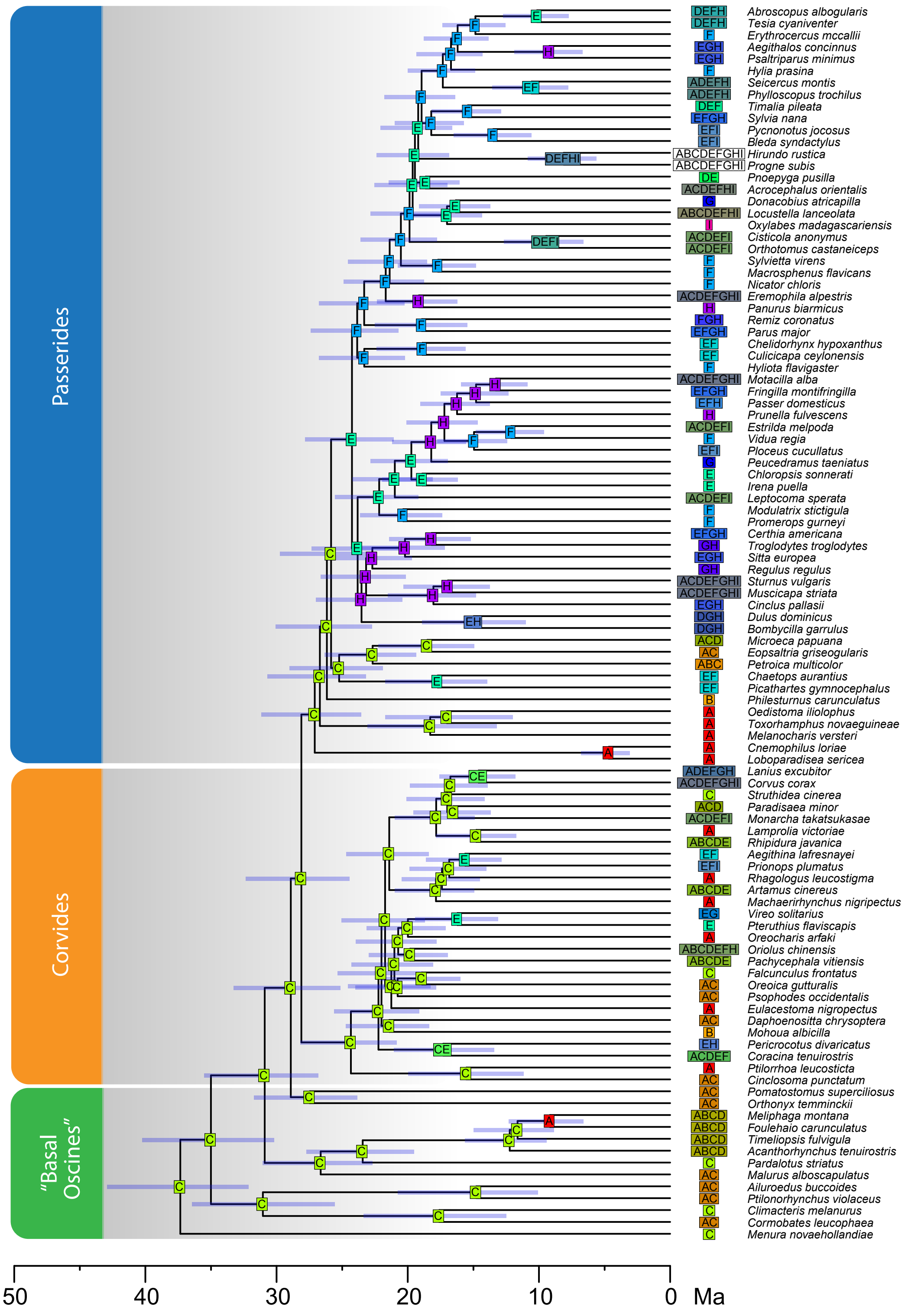

Supplementary Figure 6. Ancestral range estimation with BioGeoBEARS using alternative tree derived from calibrations from Prum et al. 2015. Analysis was performed with full distribution of clades under DEC+J model with New Guinea ancestral area not allowed before 15 Ma. Biogeographic areas: New Guinea [A], New Zealand [B], Australia [C], Wallacea [D], S and SE Asia [including Philippines; E], sub-Saharan Africa [F], New World [G], Palearctic [including N. Africa; H], and, Madagascar [I]. Bars indicate 95% highest posterior density of node date estimates.

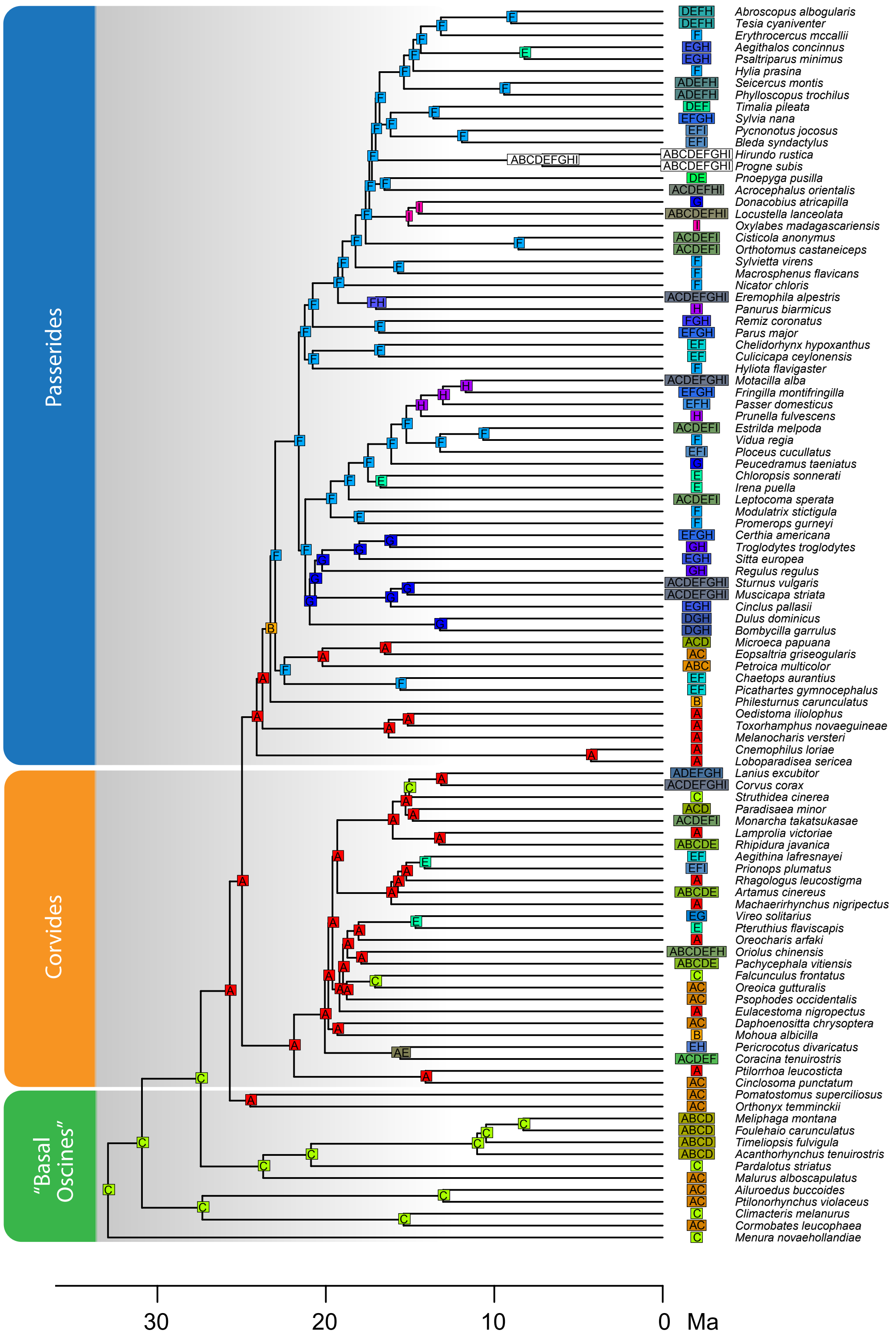

Supplementary Figure 7. Ancestral range estimation with BioGeoBEARS using full distribution of clades under DEC+J model with no area constraint. Biogeographic areas: New Guinea [A], New Zealand [B], Australia [C], Wallacea [D], S and SE Asia [including Philippines; E], sub-Saharan Africa [F], New World [G], Palearctic [including N. Africa; H], and, Madagascar [I].

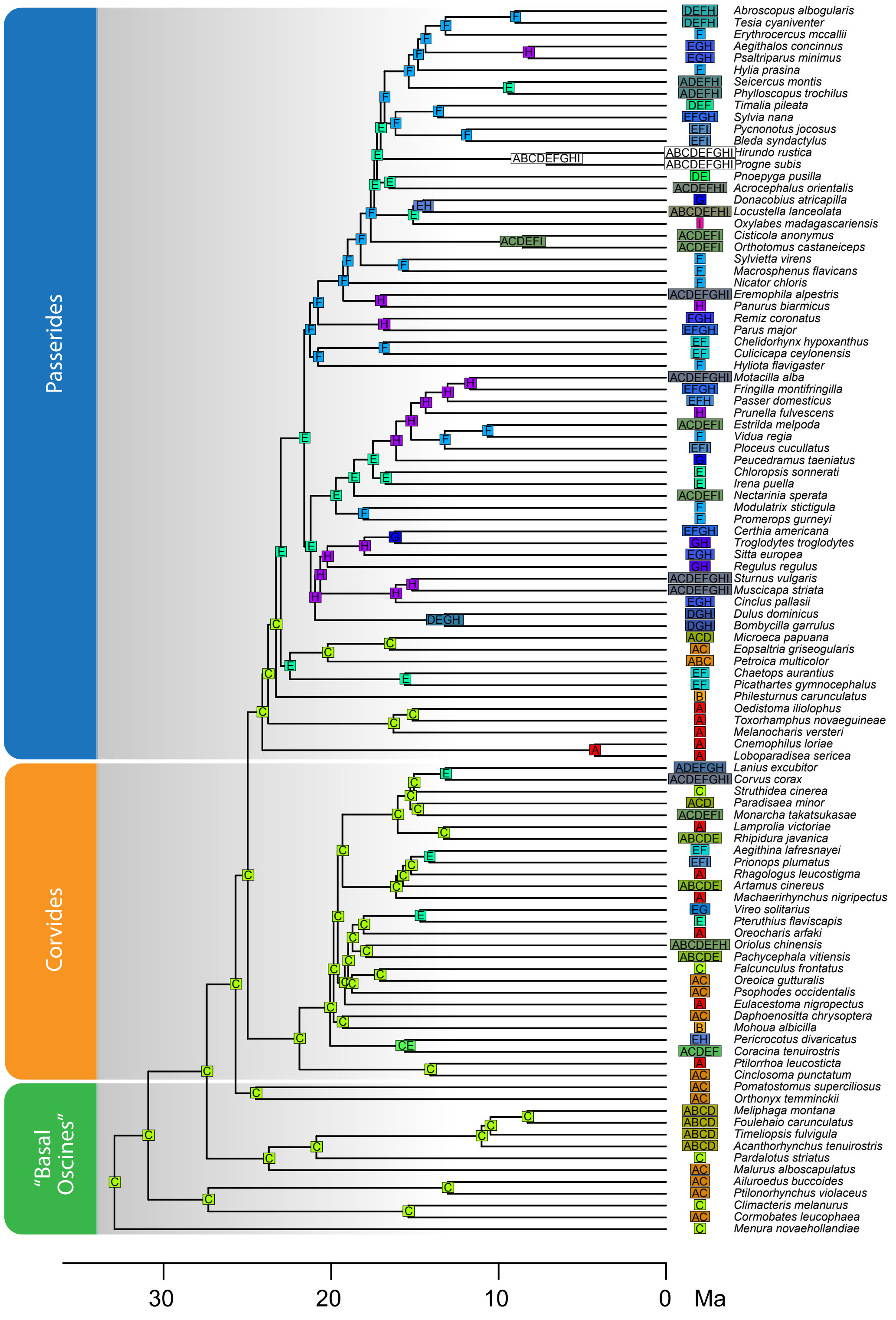

Supplementary Figure 8. Ancestral range estimation with BioGeoBEARS using full distribution of clades under DIVALIKE+J model with New Guinea ancestral range not allowed before 15 Ma. Biogeographic areas: New Guinea [A], New Zealand [B], Australia [C], Wallacea [D], S and SE Asia [including Philippines; E], sub-Saharan Africa [F], New World [G], Palearctic [including N. Africa; H], and, Madagascar [I].

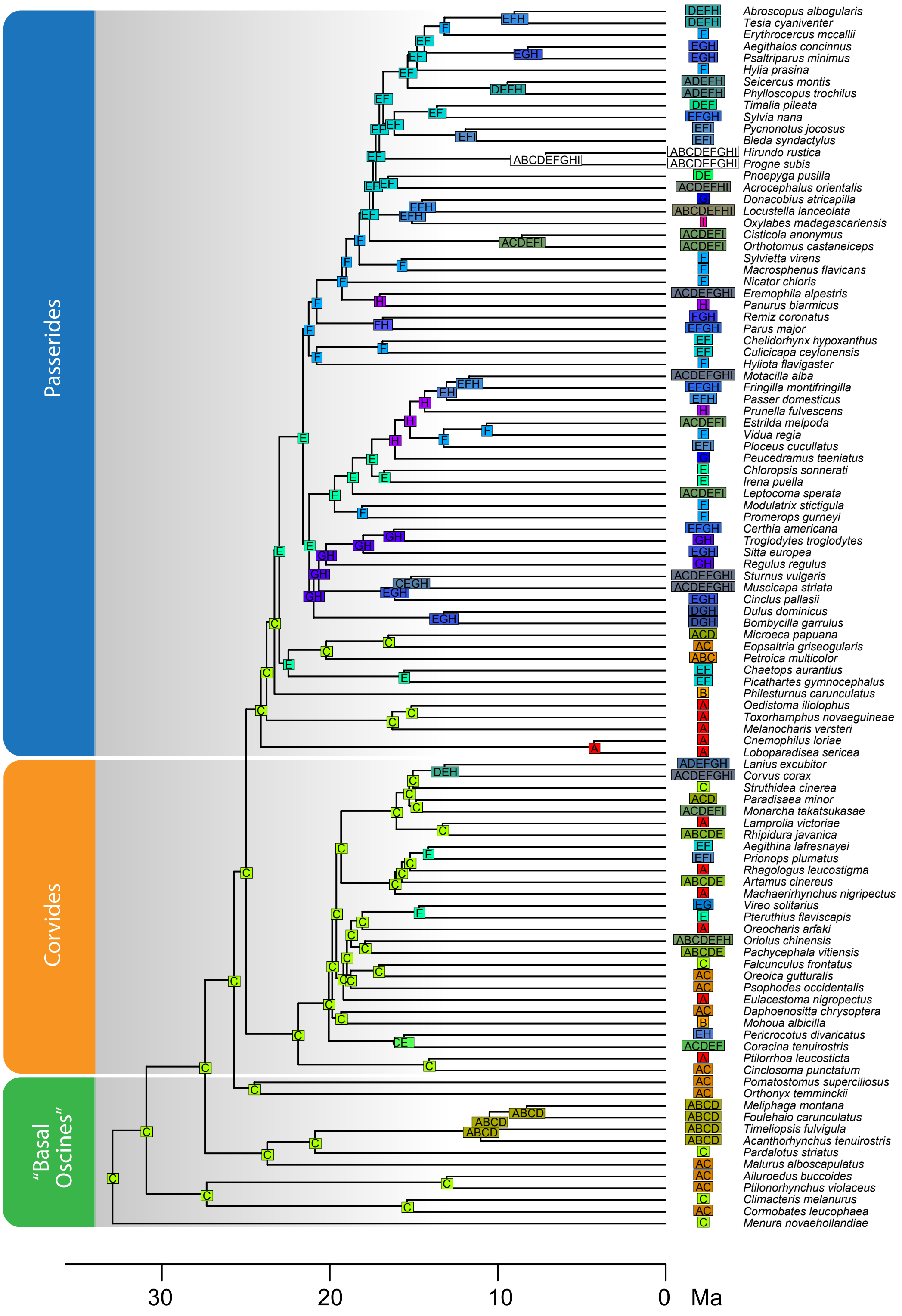

Supplementary Figure 9. Ancestral range estimation with BioGeoBEARS using full distribution of cladesunder BAYAREALIKE+J model with New Guinea ancestral range not allowed before 15 Ma. Biogeographic areas: New Guinea [A], New Zealand [B], Australia [C], Wallacea [D], S and SE Asia [including Philippines; E], sub-Saharan Africa [F], New World [G], Palearctic [including N. Africa; H], and, Madagascar [I]

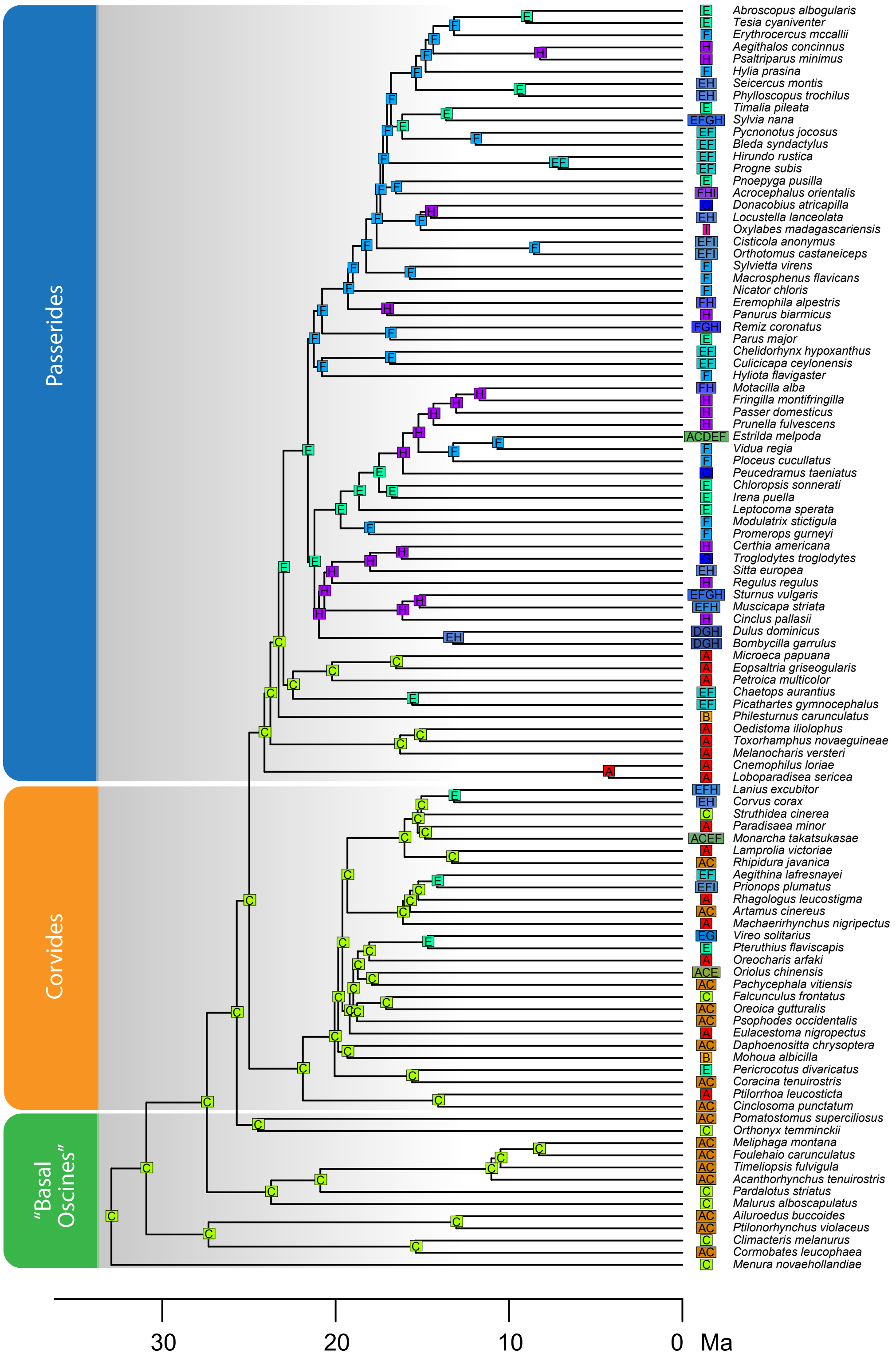

Supplementary Figure 10. Ancestral range estimation with BioGeoBEARS using inferred origin of clades under DEC+J model with New Guinea ancestral range not allowed before 15 Ma. Biogeographic areas: New Guinea [A], New Zealand [B], Australia [C], Wallacea [D], S and SE Asia [including Philippines; E], sub-Saharan Africa [F], New World [G], Palearctic [including N. Africa; H], and, Madagascar [I].

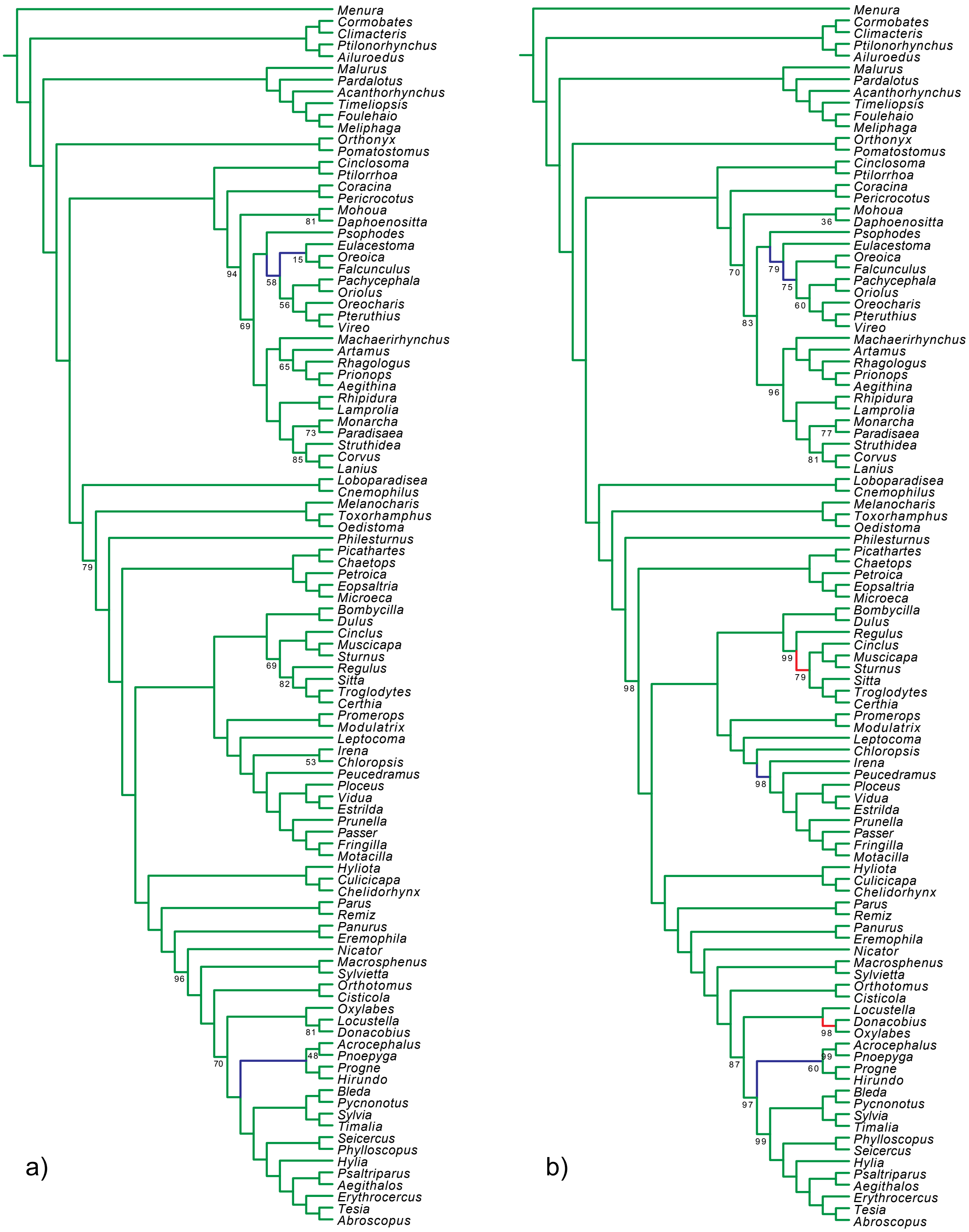

Supplementary Figure 11. Phylogenetic relationships among oscines estimated using maximum likelihood with RAxML represented as cladograms based on: a) 3839 UCE loci that do not overlap with chicken protein-coding genes, and b) the incomplete matrix coded as purines and pyrimidines. Bootstrap support values are 100% unless otherwise indicated by numbers below nodes. Red branches indicate conflicting relationships that are highly supported in both the tree and the maximum likelihood topology inferred from the incomplete matrix (Supp. Fig. 1); blue branches indicate conflicting relationships that are not well-supported in either the tree or the maximum likelihood tree inferred from the incomplete matrix (Supp. Fig. 1).

Supplementary Tables

Supplementary Table 1. Ancestral area estimation comparison. Results of ancestral area estimation with BioGEOBEARS under alternative area coding schemes, area constraints, and biogeographic models. Log likelihoods (LnL), parameter estimates (d = dispersal rate; e = extinction rate; j = jump/founder-event speciation rate), and the Akaike Information Criterion (AIC) are shown. In all comparisons, models featuring the +j parameter were a better fit according to AIC.

Area coding	Area constraint	Biogeographic Model	LnL	No. of Para-meters	d	e	j	AIC
Clade full distribution	None	DEC	-544.2919993	2	0.131400384	0.123329809	-	1092.583999
Clade full distribution	None	DEC+J	-528.6732971	3	0.103637963	1.00E-12	0.01688541	1063.346594
Clade full distribution	None	DIVALIKE	-565.4029502	2	0.120182614	0.277078875	-	1134.8059
Clade full distribution	None	DIVALIKE+J	-532.5746137	3	0.108907772	1.00E-12	0.014450084	1071.149227
Clade full distribution	None	BAYAREALIKE	-483.3219584	2	0.047713772	0.249787512	-	970.6439169
Clade full distribution	None	BAYAREALIKE +J	-466.3552156	3	0.051700667	0.091138618	0.011785616	938.7104312
Clade origin	None	DEC	-391.1692546	2	0.048852868	0.054179743	-	786.3385091
Clade origin	None	DEC+J	-362.2800311	3	0.037835844	1.00E-12	0.023833139	730.5600623
Clade origin	None	DIVALIKE	-397.4694434	2	0.053317122	0.043459414	-	798.9388868
Clade origin	None	DIVALIKE+J	-365.4133237	3	0.039545752	1.00E-12	0.021181363	736.8266475
Clade origin	None	BAYAREALIKE	-334.5106556	2	0.014200099	0.370286656	-	673.0213113
Clade origin	None	BAYAREALIKE +J	-323.6850372	3	0.016332134	0.150878001	0.015600018	653.3700744
Clade full distribution	NG not allowed until 15 Ma	DEC	-506.2907659	2	0.711457737	0.644555593	-	1016.581532
Clade full distribution	NG not allowed until 15 Ma	DEC+J	-474.4742296	3	0.445973451	0.125331802	0.070592683	954.9484593
Clade full distribution	NG not allowed until 15 Ma	DIVALIKE	-535.3374764	2	0.721592752	0.926748808	-	1074.674953
Clade full distribution	NG not allowed until 15 Ma	DIVALIKE+J	-472.961273	3	0.455220043	0.114379615	0.066278442	951.9225459
Clade full distribution	NG not allowed until 15 Ma	BAYAREALIKE	-464.1330838	2	0.349147347	0.271151328	-	932.2661675
Clade full distribution	NG not allowed until 15 Ma	BAYAREALIKE +J	-444.5458335	3	0.327167665	0.103364073	0.07203709	895.0916669
Clade origin	NG not allowed until 15 Ma	DEC	-402.6531624	2	0.125853091	0.061071544	-	809.3063248
Clade origin	NG not allowed until 15 Ma	DEC+J	-367.3593074	3	0.167763972	0.126837421	0.084607027	740.7186148
Clade origin	NG not allowed until 15 Ma	DIVALIKE	-424.3742855	2	0.128864809	0.417048461	-	852.748571
Clade origin	NG not allowed until 15 Ma	DIVALIKE+J	-368.433838	3	0.171331409	0.119036592	0.076647814	742.8676761
Clade origin	NG not allowed until 15 Ma	BAYAREALIKE	-378.6732271	2	0.141160997	0.440416295	-	761.3464543
Clade origin	NG not allowed until 15 Ma	BAYAREALIKE +J	-355.7460358	3	0.132838557	0.125571944	0.082020975	717.4920716

Supplementary Discussion

Comparison of analytical methods

For ML analysis, *a posteriori* calculation of autoMRE indicated that bootstrapping converged after 50 replicates. In Bayesian analyses, the average standard deviation of split frequencies (ASDSF) dropped below 0.01 quickly (<< 1 million generations) and remained at this level until runs were terminated (after a minimum of 5 million generations with ASDSF below 0.01). After adjusting run settings (see Methods), chains swapped frequently (adjacent chains ~0.2–0.5), but topology proposals were rarely accepted (< 0.01). Likelihood values from four independent runs plateaued quickly and stabilized in the same range. All potential scale reduction factor (PSRF) values were close to one (0.99 < PSRF > 1.01) and all effective sample size values were greater than 200. Rapid convergence among independent runs and the rarity of successful topology proposals were likely caused by the strong phylogenetic signal from such a large matrix. We removed the first 25% of generations as burn-in and summarized the remaining runs in a majority rule consensus tree.

Analyses of the incomplete matrix (ML, Bayesian, and SVDquartets) produced highly concordant results, and most nodes received strong support (Supplementary Figs. 1–2). Only a single node differed between ML and Bayesian analyses (Supplementary Fig. 1; see below). The species tree estimated by SVDquartets matched that of the concatenated analyses more closely than gene tree-based coalescent methods (GCM; Supplementary Fig. 2). Most conflicts between SVDquartets and concatenated methods involved nodes with low support in one or both methods. However, the relationships of three taxa (*Paradisaea*, *Artamus*, and *Regulus*) were strongly supported and differed from ML/Bayesian inference. All three of these relationships involved short internodes. Other discrepancies recovered in SVDQuartets, such as the placement of *Mohoua* and *Daphoenositta*, were weakly supported. Notably, relationships of major clades and deep lineages were congruent between SVDQuartets and concatenation methods.

Maximum likelihood analysis of the complete matrix produced a phylogeny mostly consistent with ML analysis of the incomplete matrix. As might be expected considering the amount of data in each matrix (4155 loci in the incomplete matrix vs. 515 loci in the complete matrix), some nodal support was lower with the complete matrix. However, two relationships with low or marginal support in the analysis of the incomplete matrix were resolved differently, but with higher support, with the complete matrix. The relationships of *Psophodes* and *Eulacestoma* were unresolved with the incomplete matrix, but these genera were inferred as sister taxa with 85% bootstrap support with

the complete matrix. Analysis of the complete matrix resulted in *Regulus* as sister to the clade comprising *Sitta*, *Troglodytes*, and *Certhia* with 71% bootstrap support. Conversely, analysis of the complete matrix resulted in *Regulus* as sister to *Dulus*+*Bombycilla* with 85% bootstrap support. SVDQuartets analysis produced a third, moderately supported relationship for *Regulus* (Supplementary Fig. 2). Thus, the relationships of *Regulus* are best considered unresolved. The last discrepancy between analysis of the complete and incomplete matrices involved a trio of genera (*Locustella*, *Donacobius*, and *Oxylabes*) that produced conflicting relationships in all analyses (see below).

ML analysis of the 3839 loci that did not contain protein-coding sequences recovered a topology identical to that obtained when we included all loci with the exception of three nodes that were not well-supported (Supplementary Fig. 11a). Likewise, we obtained a similar topology from the "RY-coded" analysis as in our original analyses with the exception of 5 nodes, 3 of which were not well-supported (Supplementary Fig. 11b). In these two additional analyses, the minor conflicts do not affect our biogeographic conclusions.

Gene tree-based coalescent methods (GCMs) produced species trees with much lower support than concatenated methods and SVDquartets (Supplementary Fig. 3). We note two important patterns in the species-tree results of GCMs – strong conflict among species tree methods and a spurious, but predictable, placement of taxa that had shorter average sequence lengths. For example, the placement of *Mohoua* differed markedly between concatenated analyses and GCMs. Whereas concatenated analyses yielded strong support for *Daphoenositta* and *Mohoua* as sister taxa and embedded well within the Corvides, all GCMs placed *Mohoua* in a more basal position as sister to the Corvides, sister to the Passerides, or even sister to the Corvides+Passerides. DNA for *Mohoua* was extracted from the toepad of a museum study skin, rather than from fresh tissue which we used for all the other species. Although we recovered many UCE loci from the toe pad extraction, the sequences were notably shorter than those from other samples (Supplementary Data 1). Samples derived from fresh tissue that had shorter average locus lengths (e.g. *Eulacestoma, Modulatrix,* and *Macrosphenus*) also had anomalous placements in species trees (Supplementary Fig. 3). We hypothesized that missing data was causing this discrepancy in phylogenetic placement, so we artificially shortened the sequences of other taxa that had strong relationships in both concatenated and species-tree analyses (Python code by C Oliveros). Shorter sequences had no effect on concatenated analyses, but they substantially altered results in summary species tree methods; taxa with shortened sequences were inferred to originate earlier in their clade, or even sister to the whole clade (Supplementary Fig. 4). All disagreement between concatenated

and GCM analyses involved A) sequence length disparity, B) weakly-supported nodes, or C) conflict among summary-species tree methods. Because none of the concatenated results were unambiguously contradicted by GCM analyses, for the remainder of the paper, we refer to the concatenated results.

All analyses produced conflicting results regarding the relationships among a trio of taxa: *Locustella lanceolata*, *Donacobius atricapillus*, and *Oxylabes madagascariensis* (Supplementary Figs. 1–3). Maximum likelihood analysis of the concatenated, incomplete matrix produced strong support for *Oxylabes* as sister to the other two species, whereas Bayesian and SVDQuartets analysis of the same matrix, and ML analysis of the complete matrix, produced strong support for *Locustella* as sister to the other two species. This discrepancy was the only difference between Bayesian and ML analysis of the incomplete matrix. Three of the four GCMs (STAR, STEAC, and ASTRAL) produced strong support for the third possible topology, with *Donacobius* sister to the other two species (Supplementary Fig. 3). The fourth GCM (MP-EST) recovered a sister relationship between *Locustella* and *Oxylabes*, but *Donacobius* was more distantly related to the pair. Previous studies with greater taxon sampling within these groups, but using fewer loci, produced a variety of moderately supported results. Oliveros et al.[1] found *Donacobius* sister to Locustellidae+Bernieridae (our GCM results), whereas Alström et al.[2] and Johansson et al.[3] recovered Locustellidae sister to *Donacobius*+Bernieridae (our Bayesian and SVDquartets results). Notably, the results in Alström et al.[2] were only supported by one of the five loci examined.

Discussion of phylogenetic relationships

Below, we restrict our discussion of phylogenetic relationships to the concatenated results (Fig. 2, Supplementary Fig. 1), except where noted. We use higher level classification names of Cracraft[4] unless noted otherwise.

Our results at the base of the oscine phylogeny largely mirror previous studies[5,6] with Menuridae (*Menura*), Climacteridae+Ptilonorhynchidae (*Cormobates*, *Climacteris*, *Ptilonorhynchus*, and *Ailuroedus*), and Meliphagoidea (*Malurus*, *Pardalotus*, *Acanthorhynchus*, *Timeliopsis*, *Foulehaio*, and *Meliphaga*) branching in succession. Claramunt and Cracraft[7] recovered a novel relationship within Meliphagoidea, with *Meliphaga* sister to *Malurus*+*Pardalotus*, but that is contradicted by our results and other studies[5,8]. We find strong support for the sister pair of *Orthonyx* and *Pomatostomus* as sister to the remainder of the oscines, in agreement with Claramunt and Cracraft[7]. Previous studies have often recovered *Orthonyx* and *Pomatostomus* branching sequentially[5,9], whereas others have found the sister relationship we recovered[6,10–12]. The next branch subtends a well-supported split into two large clades:

the Corvides and Passerides. Basal relationships within these two clades differ from all previous studies.

Infraorder Corvides. The Corvides are subtended by a long branch and strongly supported as monophyletic. At the base of the Corvides we recover strong support for the sister pairing of *Cinclosoma* and *Ptilorrhoa* as sister to all other Corvides. Previous studies have found *Cinclosoma* and/or *Ptilorrhoa* embedded well within the Corvides[5,9–12], except for Selvatti et al.[6], which found the same relationship as the current study. Claramunt and Cracraft[7] recovered a very different arrangement of taxa at the base of Corvides, with several putative passeridan lineages (e.g., Picathartidae, Melanocharitidae, and *Philesturnus*) branching sequentially from the base of the clade. The remaining Corvides are composed of five main clades separated by extremely short internodes. These short internodes likely caused the disparate relationships within Corvides among ours and previous studies. We found strong support for the sequential branching of Campephagidae (*Coracina*+*Pericrocotus*), then *Mohoua*+*Daphoenositta*, and three large clades. The campephagids, *Mohoua*, and *Daphoenositta* have previously been placed in a variety of relationships in the Corvides. For example, Aggerbeck et al.[11] and Jønsson et al.[12] found *Mohoua* sister to all other Corvides, but not sister to *Daphoenositta*, which was embedded in a subclade within the Corvides. Selvatti et al.[6] placed these genera far apart in different subclades of the Corvides, Jønsson et al.[9] placed them both in unresolved positions at the base of the Corvides, and Jetz et al.[10] placed them embedded within different parts of the Corvides, but with equivocal support. Claramunt and Cracraft[7] found *Mohoua* and *Daphoenositta* sister to *Melanocharis* and *Vireo*, respectively, which our results place far apart in the oscine radiation.

We find strong support for three large clades in Corvides: a novel clade we call superfamily Orioloidea, the whistlers and allies; Malaconotoidea, the shrike-like birds; and Corvoidea, the crows and allies (Supplementary Fig. 1). We recover *Eulacestoma, Psophodes, Oreoica, Falcunculus, Pachycephala, Oriolus, Oreocharis, Pteruthius*, and *Vireo* in a strongly supported clade from Bayesian, maximum likelihood, and SVDQuartets analyses (Supplementary Figs. 1–2). Malaconotoidea and Corvoidea are subtended by long internodes, whereas the Orioloidea have an extremely short basal branch and many short internodes within the clade. Aggerbeck et al.[11] also found support for three clades within their “Core-Corvoidea,” however, their placement of several lineages differed from our results. For example, they found Campephagidae (*Coracina*) was sister to the rest of the shrike-like birds (their clade Y), but we found strong support for the placement of Campephagidae outside the Malaconotoidea. Aggerbeck et al.[11] also found support for a clade similar to our Orioloidea (their clade X), but with additional taxa inside (e.g., *Cinclosoma* and *Daphoenositta*) that strongly conflict with our results. Jønsson et al.[9] recovered a weakly-supported clade comprising

fewer members than our Orioloidea. Instead, they found equivocal support at the base of their "Core-Corvoidea" for taxa such as *Eulacestoma*, *Oreocharis*, and *Psophodes*. The Corvides topology of Jetz et al.[10] differs dramatically from our study (Supplementary Fig. 5). For example, they recovered a clade of mostly Corvides taxa that also contained taxa such as Callaeidae (*Philesturnus*), Cnemophilidae, and Melanocharitidae, which we recovered as basal Passerides (see below). Furthermore, none of our major Corvides clades (e.g., Orioloidea, Malaconotoidea, and Corvoidea) are supported by Jetz et al.[10] Instead, they recovered taxa within our Orioloidea (Supplementary Fig. 1) scattered throughout their Corvides clade (Supplementary Fig. 5).

Infraorder Passerides. The Passerides form a clade comprising approximately one-third of extant avian diversity, and relationships within this clade are notoriously difficult to resolve[5,6,13]. Several lineages branch sequentially from the base of Passerides. These lineages have all been lumped into a grouping called "transitional oscines" by previous authors[6,9,11] and include, Cnemophilidae (*Loboparadisea*+*Cnemophilus*), Melanocharitidae (*Melanocharis*, *Toxorhamphus*, and *Oedistoma*), and Callaeidae (*Philesturnus*). Our results support an expanded Passerides that includes these aforementioned basal lineages because this clade is well supported across all analytical methods. We follow the parvorder names of Cracraft[4] to identify seven higher-level clades within the Passerides (Supplementary Fig 1), Cnemophilida, Melanocharitida, Eupetida, Petroicida, Muscicapida, Passerida, Sylviida. We sampled only one genus from Callaeidae (*Philesturnus*), so we refrain from identifying a parvorder for this group. The next branch subtends a clade comprising sister taxa Picathartidae+Chaetopidae (Parvorder Eupetida), which is sister to Parvorder Petroicida (*Petroica*, *Eopsaltria*, and *Microeca*). Our finding of Eupetida sister to Petroicida is unique among recent studies[5–7,9–11].

The next clade is subtended by a long branch and comprises the major groups of Passerides: Parvorders Muscicapida, Passerida, and Sylviida; the latter is sister to Muscicapida+Passerida. This clade is informally referred to as the "Core-Passerides" (Fig. 1, Supplementary Fig. 1). Muscicapida contains four successive sister lineages: Superfamilies Bombycilloidea (*Bombycilla* and *Dulus*), Muscicapoidea (*Cinclus*, *Muscicapa*, and *Sturnus*), Reguloidea (*Regulus*) and Certhioidea (*Sitta*, *Troglodytes*, and *Certhia*), but see above for discussion of alternative placements of *Regulus* within Muscicapida.

The first branch within Passerida subtends two African lineages, Promeropidae (*Promerops*)+Arcanatoridae (*Modulatrix*). The Nectarinidae (*Leptocoma*) are sister to a pair of SE Asian families, Irenidae (*Irena*) and Chloropseidae (*Chloropsis*). The remainder of this clade comprises what Cracraft[4] called "'core' passeridans," which

includes the monotypic Peucedramidae (*Peucedramus*) through *Fringilla*+*Motacilla*. *Peucedramus* is the earliest diverging lineage, followed by a diverse clade of Old World taxa comprising ploceid weavers and viduid and estrildid finches—Ploceoidea, sensu Cracraft[4]. This clade is sister to a clade composed of Prunellidae (*Prunella*), Passeridae (*Passer*), Fringillidae (*Fringilla*), and Motacillidae (*Motacilla*).

Parvorder Sylviida is the third major clade of Passerides. The first branch subtends a novel sister relationship: Hyliotidae (*Hyliota*)+Stenostiridae (*Culicicapa* and *Chelidorhynx*). Previous studies placed *Hyliota* as an unresolved lineage near the base of the Passerides[3,14]. Analysis of full mitochondrial genomes[15] found moderate support for a sister relationship between *Hyliota* and *Poecile*, but total sampling was sparse and Stenostiridae was not sampled. The next series of sequentially sister lineages are represented by a clade of Paridae (*Parus*)+Remizidae (*Remiz*), followed by Panuridae (*Panurus*)+Alaudidae (*Eremophila*); the latter clade is referred to as Superfamily Alaudoidea by Cracraft[4]. The next two branches represent African radiations: Nicatoridae (*Nicator*) followed by Panuridae (*Panurus*)+Alaudidae (*Eremophila*); the latter clade is referred to by Cracraft[4] as Superfamily Alaudoidea. Remaining lineages in Sylviida have received considerable attention with little consensus of branching pattern[13]. Here, we found high support for many relationships; however, short internodes left some relationships along the backbone equivocal. For example, relationships among four lineages were equivocal, including branches subtending 1) Cisticolidae (*Cisticola* and *Orthotomus*); 2) Bernieridae (*Oxylabes*), Locustellidae (*Locustella*), and Donacobiidae (*Donacobius*), but see above for alternative topologies in this clade; 3) Acrocephalidae (*Acrocephalus*) and Pnoepygidae (*Pnoepyga*); and 4) Hirundinidae (*Progne* and *Hirundo*). Finally, a large and diverse clade was recovered. Within this clade, we found support for a major split between Pycnonotidae (*Bleda* and *Pycnonotus*), Timaliidae and allies (*Timalia*), and Sylviidae (*Sylvia*) from Phylloscopidae (*Phylloscopus* and *Seicercus*), Hyliidae (*Hylia*), Aegithalidae (*Psaltriparus* and *Aegithalos*), Erythrocercidae (*Erythrocercus*) and Cettiidae (*Tesia* and *Abroscopus*).

Jetz et al.[10] used a hybrid super-tree/super-matrix approach with topological constraints to reconstruct a phylogeny of all bird species, which subsequently formed the phylogenetic basis of several influential analyses of bird evolution[16–20]. Because Jetz et al.[10] included all bird species, our results can be compared directly to their phylogeny. We computed a consensus tree from 1,000 trees with the "Hackett" constraints downloaded from birdtree.org/subsets, limiting the species to those we included in our analysis (Supplementary Fig. 5). Many strongly supported differences are apparent at all levels of the oscine phylogeny, especially in Corvides, placement of transitional Oscine lineages, and basal relationships in Passerides. To quantify concordance between the two trees, we calculated the normalized Robinson-Foulds metric[21] in Paup, ver. 4.0a146[22], with a result of 0.438. This value means that ~44% of the bipartitions

found in the two trees are unique to only one of the trees. This large discrepancy likely relates to the disparate approaches of the two studies. Jetz et al.[10] analyzed a large, sparse super-matrix from relatively few markers whereas we analyzed a massive character matrix for a limited number of samples.

Discussion of divergence time estimates

Dates derived from the Jarvis et al.[23] secondary calibrations (Fig. 1) differed slightly from those produced with the Prum et al.[24] secondary calibrations (Supplementary Fig. 6), but 95% highest posterior density intervals broadly overlapped. For example, we inferred the base of the Corvides as 21.9 Ma (CI: 19.8–24.0 Ma) with the Jarvis et al.[23] calibrations, but 24.3 Ma (CI: 20.8–28.2 Ma) with the Prum et al.[24] calibrations. Important to our discussion of biogeographic history, the two methods broadly agree about the timeframe of oscine diversification. Although we inferred some nodes as latest Oligocene with the Prum calibrations, compared to earliest Miocene with the Jarvis calibrations, the earliest inferred dispersal events out of Australasia cluster around the initial uplift of Wallacea with both sets of calibrations.

We compared these results to oft-cited rates of mitochondrial DNA evolution in birds. Sequence capture techniques produce reads from non-target regions such as the mitochondrial genome. We assembled mitochondrial genomes from cleaned reads using the program ARC (https://ibest.github.io/ARC/) using the mitochondrial genome of *Vidua chalybeata* (GenBank AF090341) as a reference. We aligned contigs of mitochondrial genomes to the annotated reference genome using Geneious ver. 6.1.2. From these alignments, we extracted gene sequences of NADH subunit 2 (ND2) and cytochrome b (cytb) for each individual. We estimated rates of mitochondrial evolution in ND2 and cytb using the calibrated ultrametric UCE topology in BEAST 2.2[25]. We selected a separate uncorrelated lognormal relaxed clock for each gene. We partitioned each mitochondrial gene by codon position, and for each partition, we selected the GTR+G model and estimated base frequencies. We executed two independent $1x10^8$ generation MCMC runs, each sampled every $1x10^5$ generations. We removed the first 25% of posterior samples as burnin, and we assessed MCMC convergence and stationarity in Tracer ver. 1.6[26]. For each gene, we then calculated the mean substitution rate estimate and its 95% highest posterior density interval from the post-burnin posterior distribution.

Using our topology and time estimates, we recovered a divergence rate of 2.3% per million years for cytochrome b (CI 2.20%–2.42%) and 3.2% per million years for ND2 (CI 3.08%–3.34%). These estimates are faster than the 2% average rate cited for birds

but fall well within the range of rates estimated empirically from a variety of avian taxa[27,28].

Discussion of ancestral range estimates

We focus discussion of biogeographic results on four important clades: all Oscines, the Corvides, the Passerides, and the Core-Passerides. Ancestral range estimates for these clades using their full distribution or their inferred origin produced almost identical results. Model selection with AIC indicated that the DEC-LIKE+*j* model was a better fit than DEC-LIKE, and therefore we present results of reconstructions using full clade distributions and the DEC-LIKE+*j* model (Fig. 2, but see examples under different modeling choices Supplementary Figs. 7–10).

Given the DEC-LIKE+*j* model, biogeographic analyses estimated the ancestral range of all oscines as Australia. An Australian origin of oscines has been consistently recovered in other studies as well[5,9,11]. The estimated ancestral ranges of Corvides and Passerides varied mainly depending on whether the 15 Ma constraint on New Guinea was used. Without the constraint, the ranges of both nodes were estimated to be New Guinea. However, when a New Guinea range prior to 15 Ma was disallowed, the ranges of both nodes were reconstructed as Australia (Fig. 1). A New Guinea origin for these clades have been found by other authors[9,11] but their results rely on two important, albeit questionable, assumptions. First, these studies assume an older age for these nodes (~30–45 Ma), which appears to be too old based on more recent and independent estimates of the timing of avian diversification[23,24]. The second assumption is that small, ephemeral proto-Papuan islands existed and were biologically relevant for ancestral range estimation. Our study is the first to estimate an Australian ancestral range for these two clades, which we believe is a more plausible alternative to the proto-Papuan origin hypothesis for these groups because of its consistency with paleogeographic reconstructions and the oscine fossil record (see below). Origin of the core-Passerides, the first major oscine clade to radiate outside of Australasia, also depended on presence or absence of the 15 Ma New Guinea constraint. With New Guinea constrained, we inferred a SE Asian origin of the core-Passerides, followed by a rapid radiation and multiple dispersals into the Palaearctic and sub-Saharan Africa. Without a constraint, the origin of this clade was reconstructed as sub-Saharan Africa.

Using alternate models (Supplementary Table 1), biogeographic reconstructions were similar to those inferred with the DEC-LIKE+*j* model. Identical to results from the DEC-LIKE+*j* model (Fig. 1), DIVA-LIKE+*j* (S7) and BAYAREA-LIKE+*j* (S8) models with New Guinea emergence constrained also inferred Australian origin of oscines, Corvides, and Passerides, as well as the SE Asian origin of Core-Passerides. The only substantial differences between models were found when analyses allowed emergence of New

Guinea prior to 15 Ma, when the BAYAREALIKE and BAYAREALIKE+J models yielded Australia+New Guinea or Australia+New Guinea+S and SE Asia for Oscines, Corvides, and Passerides. However, these models assume all cladogenesis occurs within, rather than between designated areas, an unrealistic assumption likely violated in songbirds.

Paleogeography of Wallacea and Australasia

Wallacea comprises a composite geological landscape that assembled within a boundary zone of extensive tectonic convergence between the Australian, Pacific, and Eurasian plates. As such, the geotectonic history of this ecoregion is exceptionally complex, with virtually all known surface and mantle processes locally active during the Cenozoic[29–31]. Although knowledge and understanding of this regional geodynamic complexity is far from complete, recent advances in resolving the broad-scale geological assembly of Wallacea has provided an important spatio-temporal framework for testing biogeographic hypotheses and exploring the potential role of this insular system in linking Australasian biotas with Southeast Asia and beyond[32].

Australia's separation from Antarctica was largely complete by the Late Cretaceous, yet these East Gondwanan fragments remained in close proximity well into the Paleogene[33]. Tectonics in this region shifted dramatically in the Late Eocene (~45 Ma) as seafloor spreading ended in the Tasman and Coral Seas but accelerated along the Southeast Indian Ridge (SIR), starting Australia's rapid progression north towards its present-day position[34]. This, in turn, led to subduction of oceanic lithosphere at the Java trench, which continued throughout the Oligocene, steadily reducing the vast expanse of open ocean between Australasia and Sundaland[29]. During this period, emergent land within Wallacea was limited to portions of West Sulawesi and a volcanic arc of small isolated islands to the east that remained separated from the Australian continental margin by a deep-sea passage spanning hundreds of kilometers.

Extensive land formation in Wallacea was initiated in the Early Miocene (~ 23 Ma) due to tectonic collision between proto-Sulawesi fragments and the Sula spur, an Australian continental promontory extending northwest from the Bird's Head region of present-day New Guinea[30,31]. Continued convergence at this collisional boundary resulted in widespread island formation and orogenic uplift in portions of Sulawesi, providing the first links between Australasia and Sundaland, albeit in the form of island chains and not a continuous land bridge. This increased connectivity was short-lived however, and by 15 Ma subduction rollback in the region caused much of the newly formed land to subside, decreasing land area across Wallacea[29,30,32]. Consequently, the most likely window for dispersal from Australia to SE Asia spanned roughly 23–15 Ma. This

reduction of land area during the Miocene may explain the absence of relictual oscine lineages in Wallacea that would lend support for such a colonization history.

As subsidence continued in Wallacea and extensional deformation began fragmenting the Sula spur into the Banda embayment during the mid-Miocene, bulldozing of pre-collisional complexes along the Australian and Pacific tectonic boundary promoted the development of small subaerial islands in the vicinity of present-day New Guinea around 15 Ma[35,36]. Considerable debate and uncertainty remain with respect to the timing and geotectonic processes that drove New Guinea's rapid and complex orogenesis, but paleogeographic models generally agree that only ephemeral, low-lying islands and carbonate platforms existed in the region until uplift of the Central Dividing Ranges (CDR) began in the Late Miocene[35] or Pliocene[37], with the possible exception of limited early uplift in the Papuan Peninsula during the Oligocene[35,36]. The latter hypothesis for an early Papuan Peninsula orogeny appears exceedingly unlikely, as there is no biogeographic evidence to support such an early terrestrial history in the region[38]. van Ufford's[36] model for development of the CDRs suggests uplift initiated around 12 Ma due to under-thrusting of Australian continental basement and bulldozing of passive-margin strata, followed by early stage collisional orogenesis at about 8 Ma that gave rise to much of the extensive New Guinea highlands by 5 Ma. This hypothesis differs substantially from that of Hill and Hall[37], who posit that New Guinea remained largely submerged until about 5 Ma, when a shift in tectonic motions initiated convergence between the Australian and Pacific plates, leading to rapid uplift of the fold-and-thrust montane belt that comprises the CDRs. Despite the profound differences between these competing tectonic models, it is now clear that New Guinea's remarkably recent geological development and rapid orogeny largely precludes the island from playing a significant role in early Oscine diversification and dispersal out of Australia.

Paleontological context

Although the oscine fossil record is sparse, and few taxa are placed phylogenetically, our timeframe and biogeographic hypothesis for oscine diversification agrees broadly with Mayr's[39] review of paleontological constraints on passerine evolution. Our data indicate that crown oscine lineages first reached Asia at the Oligocene-Miocene transition and dispersed worldwide shortly thereafter. The earliest oscine fossils from the northern hemisphere are from late Oligocene Europe[40,41], but they have not been identified as either crown or stem lineages. The earliest passerine fossils from Africa[42] and the New World[43,44] are from the Miocene.

Paleoecology and evolution of the Australasian mesic biota

We propose that oscine songbirds initially diversified in isolation on the Australian plate, with dispersive elements subsequently colonizing Southeast Asia and other regions of the globe in the Early Miocene when tectonic collision and uplift in Wallacea produced newly emergent islands that enabled biotic interchange between Australasia and Sundaland. Although unconstrained biogeographic reconstructions indicate that many of these early oscine lineages originated in New Guinea (Supplementary Fig. S7), we interpret this result as a bias associated with the severe Miocene aridification of Australia and wholesale reduction of its mesic biota[45], as the New Guinea region largely remained submerged until the Late Miocene or Early Pliocene[35,37].

Cool subtropical environments persisted across much of the Australian continent during the Eocene to Early Oligocene, harboring diverse rainforest communities with strong Gondwanan affinity. As Australia drifted to warmer latitudes, its climate gradually shifted and periods of drought became more pronounced, resulting in the first signs of contraction among rainforest habitats by the Late Oligocene[46,47]. Widespread aridification intensified around the mid-Miocene climatic optimum, driving Australia's once extensive mesic biome into small refugia along the eastern coast, further compounding the sharp decline and extinction of rainforest-adapted taxa that was already underway[45,48]. Importantly, the concomitant development of New Guinea's emerging highland landscape in the Late Miocene provided a vast new refuge for these relictual lineages to colonize along Australia's northern continental margin, which likely prevented extinction of numerous temperate and subtropical groups. Thus, in many respects, the rich montane biota that now inhabits the New Guinea highlands provides a unique window to Australia's past, and some of the early ancestral Gondwanan lineages that once characterized its subtropical rainforest environments. We suggest that this regional paleoclimatic history likely explains the presence of early oscine lineages in New Guinea that predate the island's recent geological development. Prime examples of lineages exhibiting long and bare branches indicative of this relictual hypothesis include the New Guinea endemic Satinbirds (Cnemophilidae) and Berrypeckers (Melanocharitidae), as well as several endemic monotypic genera within the Corvidan radiation (*Eulacestoma*, *Oreocharis*, and *Rhagologus*), all montane taxa that arose prior to the emergence of New Guinea's central cordillera.

Although this hypothesis differs markedly from recent studies of oscine diversification[5,9,11,12], our results strongly corroborate an earlier hypothesis developed by Schodde[49] that has largely been overlooked because of incongruent temporal frameworks or unconstrained biogeographic analyses. Schodde and colleagues[49–51] examined the distribution and community composition of Australasian avifaunae in the context of regional tectonic history, paleoecology, and phylogeny. They identified an

ancestral “Tumbunan” avifauna endemic to subtropical montane forests of New Guinea and a narrow corridor of rainforest refugia along the northeastern Australian coast. This community is diverse and elevationally structured within New Guinea, but comparatively depauperate in Australia. Schodde[49] hypothesized that aridification of Australia likely depleted the once widespread wet-adapted communities, which subsequently found refuge in New Guinea during its rapid orogenesis in the Late Miocene to Early Pliocene. Schodde and Christidis[51] questioned the hypothesis that New Guinea comprises the area of initial diversification of Corvides[9,11,12], citing geological, temporal, and distributional inconsistencies, and concluded that New Guinea was more likely a refuge for relictual lineages as opposed to a “launch pad” for the corvoid radiation.

This signal of impoverishment and extinction among some early oscine lineages is part of a larger biogeographic pattern that is widely manifest in the Australasian mesic biota, suggesting a number of non-avian groups were similarly impacted by the mid-Miocene aridification of Australia. Byrne et al.[45] documented the decline of Australia’s mesic biota throughout the Neogene, with the most extensive extinctions and range restrictions occurring in taxa associated with temperate and subtropical rainforest environments. The palynological record for Australia provides some of the clearest evidence of this dramatic and large-scale biotic restructuring[45,47]. For example, the *Nothofagus* subgenus *Brassospora* was once widespread among Australian subtropical rainforest environments during the Eocene to Early Oligocene. Macrofossils from *Brassospora* have been recovered in Australia that date to the Oligocene, and *Brassospora* pollen has been recorded on the continent as recently as 2 Ma. Although this drought-sensitive clade is now extinct in Australia, several *Brassospora* species remain a central component of humid mid-montane forests throughout New Guinea. A similar pattern is seen in some Australasian conifers (Podocarpaceae), with *Dacrydium* and *Dacrycarpus* formerly common among subtropical environments in Australia, and members of the later genus persisting until the late Pliocene along the southern coast[52]. Both genera are now ubiquitous across the New Guinea highlands.

The fossil record for Australasian mammals is far more extensive than that for birds, and highlights several robust examples of wet-adapted marsupial clades that went extinct in Australia during the Miocene or Early Pliocene, but have persisted in New Guinea’s extensive rainforest habitats[48,53]. The Dactylopsine possums (Petauridae) were once thought to originate in New Guinea, which comprises their present-day center of diversity, but fossils of an undescribed species of *Dactylopsila* from the Late Oligocene to mid Miocene deposits at the Riversleigh site in Queensland now indicate an Australian origin for the group[54]. The Phalangerids (cuscuses, brush-tailed possums, and scaly-tailed possums: Phalangeridae) are the most diverse of the Australasian possum families with 19 of the 23 species present in the New Guinea region. Although the distribution of present-day diversity suggests that the clade arose in New Guinea,

the presence of multiple cuscus species and at least one scaly-tailed possum taxon from the Early Miocene deposits at Riversleigh indicate that an Australian origin is equally if not more likely[48,53]. A similar pattern is seen in the endemic forest wallabies (*Dorcopsulus* and *Dorcopsis*: Macropodidae) of New Guinea, which include two and three species respectively, yet an extinct *Dorcopsis* species has been described from a Pliocene fossil bed in western Victoria and multiple fossil specimens of a closely related forest wallaby (*Dorcopsoides fossilis*) has been described from the Late Miocene site of Alcoota in the Northern Territory[53]. We predict that as additional Miocene to Early Pliocene fossil sites are uncovered, further evidence of this general biogeographic trend within the Australasian mesic biota will become apparent in other marsupial groups such as the echymiperin and peroryctin bandicoots (Peramelidae), which are thought to have originated in Australia and secondarily diversified in the rainforests of New Guinea[55].

Supplementary References

1. Oliveros, C. H., Reddy, S. & Moyle, R. G. The phylogenetic position of some Philippine 'babblers' spans the muscicapoid and sylvioid bird radiations. *Mol. Phylogenet. Evol.* **65,** 799–804 (2012).
2. Alström, P. *et al.* Multilocus analysis of a taxonomically densely sampled dataset reveal extensive non-monophyly in the avian family Locustellidae. *Mol. Phylogenet. Evol.* **58,** 513–26 (2011).
3. Johansson, U. S., Fjeldså, J. & Bowie, R. C. K. Phylogenetic relationships within Passerida (Aves: Passeriformes): a review and a new molecular phylogeny based on three nuclear intron markers. *Mol. Phylogenet. Evol.* **48,** 858–76 (2008).
4. Cracraft, J. in *The Howard & Moore Complete Checklist of the Birds of the World. 4th Edition. Vol. 2* (eds. Dickinson, E. & Christidis, L.) xvii–xlv (2014).
5. Barker, F. K., Cibois, A., Schikler, P., Feinstein, J. & Cracraft, J. Phylogeny and diversification of the largest avian radiation. *Proc. Natl. Acad. Sci. U. S. A.* **101,** 11040–5 (2004).
6. Selvatti, A. P., Gonzaga, L. P. & Russo, C. A. D. M. A Paleogene origin for crown passerines and the diversification of the Oscines in the New World. *Mol. Phylogenet. Evol.* **88,** 1–15 (2015).
7. Claramunt, S. & Cracraft, J. A new time tree reveals Earth history's imprint on the evolution of modern birds. *Sci. Adv.* **1,** e1501005 (2015).
8. Gardner, J. L., Trueman, J. W. H., Ebert, D., Joseph, L. & Magrath, R. D. Phylogeny and evolution of the Meliphagoidea, the largest radiation of Australasian songbirds. *Mol. Phylogenet. Evol.* **55,** 1087–1102 (2010).
9. Jønsson, K. a, Fabre, P.-H., Ricklefs, R. E. & Fjeldså, J. Major global radiation of corvoid birds originated in the proto-Papuan archipelago. *Proc. Natl. Acad. Sci. U. S. A.* **108,** 2328–2333 (2011).
10. Jetz, W., Thomas, G. H., Joy, J. B., Hartmann, K. & Mooers, A. O. The global diversity of birds in space and time. *Nature* **491,** 1–5 (2012).

11. Aggerbeck, M., Fjeldså, J., Christidis, L., Fabre, P. H. & Jønsson, K. A. Resolving deep lineage divergences in core corvoid passerine birds supports a proto-Papuan island origin. *Mol. Phylogenet. Evol.* **70,** 272–285 (2014).
12. Jønsson, K. A. *et al.* A supermatrix phylogeny of corvoid passerine birds (Aves: Corvides). *Mol. Phylogenet. Evol.* **94,** 87–94 (2016).
13. Alström, P., Ericson, P. G. P., Olsson, U. & Sundberg, P. Phylogeny and classification of the avian superfamily Sylvioidea. *Mol. Phylogenet. Evol.* **38,** 381–97 (2006).
14. Fuchs, J., Fjeldså, J., Bowie, R. C. K., Voelker, G. & Pasquet, E. The African warbler genus Hyliota as a lost lineage in the Oscine songbird tree: Molecular support for an African origin of the Passerida. *Mol. Phylogenet. Evol.* **39,** 186–197 (2006).
15. Keith Barker, F. Mitogenomic data resolve basal relationships among passeriform and passeridan birds. *Mol. Phylogenet. Evol.* **79,** 313–324 (2014).
16. Ricklefs, R. E. & Jonsson, K. a. Clade extinction appears to balance species diversification in sister lineages of Afro-Oriental passerine birds. *Proc. Natl. Acad. Sci. USA* **111,** 11756–11761 (2014).
17. Thomas, D. B. *et al.* Ancient origins and multiple appearances of carotenoid-pigmented feathers in birds. *Proc. R. Soc. B Biol. Sci.* **281,** 20140806–20140806 (2014).
18. Barnagaud, J. Y. *et al.* Ecological traits influence the phylogenetic structure of bird species co-occurrences worldwide. *Ecol. Lett.* **17,** 811–820 (2014).
19. Healy, K. *et al.* Ecology and mode-of-life explain lifespan variation in birds and mammals. *Proc. Biol. Sci.* **281,** 20140298 (2014).
20. Dey, C. J., Valcu, M., Kempenaers, B. & Dale, J. Carotenoid-based bill coloration functions as a social, not sexual, signal in songbirds (Aves: Passeriformes). *J. Evol. Biol.* **28,** 250–258 (2015).
21. Robinson, D. F. & Foulds, L. R. Comparison of phylogenetic trees. *Math. Biosci.* **53,** 131–147 (1981).
22. Swofford, D. L. PAUP*. Phylogenetic analysis using parsimony (*and Other Methods). (2003).
23. Jarvis, E. D. *et al.* Whole-genome analyses resolve early branches in the tree of life of modern birds. *Science (80-.).* **346,** 1320–1331 (2014).
24. Prum, R. O. *et al.* A comprehensive phylogeny of birds (Aves) using targeted next-generation DNA sequencing. *Nature* 3–11 (2015). doi:10.1038/nature15697
25. Bouckaert, R. *et al.* BEAST 2: A Software Platform for Bayesian Evolutionary Analysis. *PLoS Comput. Biol.* **10,** 1–6 (2014).
26. Rambaut, A. & Drummond, A. J. Tracer v.1.5. at <http://tree.bio.ed.ac.uk/software/tracer/>
27. Weir, J. T. & Schluter, D. Calibrating the avian molecular clock. *Mol. Ecol.* **17,** 2321–2328 (2008).
28. Lovette, I. J. Mitochondrial dating and mixed support for the‘ 2% rule’ in birds. *Auk* **121,** 1–6 (2004).
29. Hall, R. Late Jurassic-Cenozoic reconstructions of the Indonesian region and the Indian Ocean. *Tectonophysics* **570-571,** 1–41 (2012).
30. Hall, R. & Sevastjanova, I. Australian crust in Indonesia. *Aust. J. Earth Sci.* **59,**

827–844 (2012).
31. Hall, R. & Spakman, W. Subducted slabs beneath the eastern Indonesia-Tonga region: Insights from tomography. *Earth Planet. Sci. Lett.* **201,** 321–336 (2002).
32. Hall, R. The palaeogeography of Sundaland and Wallacea since the Late Jurassic. *J. Limnol.* **72,** 1–17 (2013).
33. White, L. T., Gibson, G. M. & Lister, G. S. A reassessment of paleogeographic reconstructions of eastern Gondwana: Bringing geology back into the equation. *Gondwana Res.* **24,** 984–998 (2013).
34. Müller, R. D., Gaina, C. & Clark, S. in *Billion-year earth history of Australia and neighbours in Gondwanaland* (eds. Veevers, J. J. & Morgan, P.) 18–28 (Gemoc Press, 2000).
35. Cloos, M., Weiland, R. J., Warren, P. Q. & McMahon, T. P. The geotectonics of subducting slab breakoff. *Geol. Soc. Am. Spec. Pap.* **400,** 1–51 (2005).
36. van Ufford, A. Q. & Cloos, M. Cenozoic tectonics of New Guinea. *Am. Assoc. Pet. Geol. Bull.* **89,** 119–140 (2005).
37. Hill, K. C. & Hall, R. Mesozoic-Cenozoic evolution of Australia's New Guinea margin in a west Pacific context. *Geol. Soc. Am. Spec. Pap.* **372,** 265–290 (2003).
38. Toussaint, E. F. a *et al.* The towering orogeny of New Guinea as a trigger for arthropod megadiversity. *Nat. Commun.* **5,** 4001 (2014).
39. Mayr, G. The age of the crown group of passerine birds and its evolutionary significance – molecular calibrations versus the fossil record. *Syst. Biodivers.* 1–6 (2013). doi:10.1080/14772000.2013.765521
40. Manegold, A. Passerine diversity in the late Oligocene of Germany: Earliest evidence for the sympatric coexistence of Suboscines and Oscines. *Ibis (Lond. 1859).* **150,** 377–387 (2008).
41. Mourer-Chauvire, C., Hugueney, M. & Jonet, P. Discovery of passeriformes in the Upper Oligocene of France. *Comptes Rendus l Acad. des Sci. Ser. II* **309,** 843–849 (1989).
42. Brunet, J. Oiseaux miocènes de Beni Mellal (Maroc); un complément à leur étude. *Notes Mémoires du Serv. géologique du Maroc* **31,** 109–111 (1971).
43. Olson, S. L. in *Avian Biology* (eds. Farner, D. S., King, J. R. & Parkes, K. C.) 79–238 (Academic Press, 1985).
44. Noriega, J. I. & Chiappe, L. M. An Early Miocene Passeriform from Argentina. *Auk* **110,** 936–938 (1993).
45. Byrne, M. *et al.* Decline of a biome: Evolution, contraction, fragmentation, extinction and invasion of the Australian mesic zone biota. *J. Biogeogr.* **38,** 1635–1656 (2011).
46. Kershaw, A. P., Martin, H. A. & McEwen Mason, J. C. in *History of the Australian Vegetation: Cretaceous to Recent* (ed. Hill, R. S.) 299–327 (Cambridge University Press, 1994).
47. Hill, R. S. *History of the Australian vegetation: Cretaceous to Recent*. (Cambridge University Press, 1994).
48. Black, K. H., Archer, M., Hand, S. J. & Godthelp, H. *Earth and Life*. (Springer, 2012). doi:10.1007/978-90-481-3428-1
49. Schodde, R. in *Evolution and biogeography of Australasian vertebrates* (eds. Merrick, J. R., Archer, M., Hickey, G. M. & Lee, M. S. Y.) 413–458 (Australian

Scientific Publishing, 2006).
50. Schodde, R. & Calaby, J. H. in *Bridge and Barrier: the Natural And Cultural History of Torres Strait* 257–300 (ANU Press, 1972).
51. Schodde, R. & Christidis, L. Relicts from Tertiary Australasia: undescribed families and subfamilies of songbirds (Passeriformes) and their zoogeographic signal. *Zootaxa* **3786,** 501–522 (2014).
52. Dettmann, M. E. & Jarzen, D. M. The Antarctic/Australian rift valley: Late cretaceous cradle of nortteastern Australasian relicts? *Rev. Palaeobot. Palynol.* **65,** 131–144 (1990).
53. Long, J. A., Archer, M., Flannery, T. & Hand, S. J. *Prehistoric mammals of Australia and New Guinea: one hundred million years of evolution*. (University of New South Wales Press, 2002).
54. Brammall, J. R. A new petauroid possum from the Oligo-Miocene of Riversleigh, northwestern Queensland. *Alcheringa* **23,** 31–50 (1999).
55. Westerman, M. *et al.* Phylogenetic relationships of living and recently extinct bandicoots based on nuclear and mitochondrial DNA sequences. *Mol. Phylogenet. Evol.* **62,** 97–108 (2012).
